# Development of a yeast-based sensor platform for evaluation of ligands recognized by the human free fatty acid 2 receptor

**DOI:** 10.1093/femsyr/foaf001

**Published:** 2025-01-17

**Authors:** Andrea Clausen Lind, Davi De Castro Gomes, Ricardo Bisquert, Jonas Mårtensson, Martina Sundqvist, Huamei Forsman, Claes Dahlgren, Florian David, Verena Siewers

**Affiliations:** Department of Life Sciences, Chalmers University of Technology, 412 96 Gothenburg, Sweden; Department of Genetics, Evolution, Microbiology and Immunology, Institute of Biology, University of Campinas, 13083-862 Campinas, São Paulo, Brazil; Department of Life Sciences, Chalmers University of Technology, 412 96 Gothenburg, Sweden; Department of Rheumatology and Inflammation Research, Institute of Medicine, The Sahlgrenska Academy, University of Gothenburg, 413 90 Gothenburg, Sweden; Department of Rheumatology and Inflammation Research, Institute of Medicine, The Sahlgrenska Academy, University of Gothenburg, 413 90 Gothenburg, Sweden; Department of Rheumatology and Inflammation Research, Institute of Medicine, The Sahlgrenska Academy, University of Gothenburg, 413 90 Gothenburg, Sweden; Department of Rheumatology and Inflammation Research, Institute of Medicine, The Sahlgrenska Academy, University of Gothenburg, 413 90 Gothenburg, Sweden; Department of Life Sciences, Chalmers University of Technology, 412 96 Gothenburg, Sweden; Department of Life Sciences, Chalmers University of Technology, 412 96 Gothenburg, Sweden; Novo Nordisk Foundation Center for Biosustainability, Technical University of Denmark, 2800 Kgs. Lyngby, Denmark

**Keywords:** biosensors, GPCR, yeast mating pathway, ligand screening, FFAR2, GPR43

## Abstract

Yeast-based sensors have shown great applicability for deorphanization of G protein-coupled receptors (GPCRs) and screening of ligands targeting these. A GPCR of great interest is free fatty acid 2 receptor (FFA2R), for which short-chain fatty acids such as propionate and acetate are agonists. FFA2R regulates a wide array of downstream receptor signaling pathways in both adipose tissue and immune cells and has been recognized as a promising therapeutic target, having been implicated in several metabolic and inflammatory diseases. While research aiming to identify ligands recognized by FFA2R for translational applications is ongoing, screening is complicated by the complex regulatory and cell-specific responses mediated by the receptor. To simplify screening towards identification of novel ligands, heterologous platforms are valuable tools that offer efficient identification of ligand activity in the absence of regulatory mechanisms. Here, we present a yeast-based sensor designed to evaluate G protein α i1-mediated FFA2R signaling, with an assay time of 3 h. We verify this platform towards the natural agonists, propionate and acetate, and show applicability towards evaluation of synthetic agonists, antagonists, and allosteric agonists. As such, we believe that the developed yeast strain constitutes a promising screening platform for effective evaluation of ligands acting on FFA2R.

## Teaser

A yeast platform was developed for evaluation of ligands modulating the human free fatty acid 2 receptor activity, in a system devoid of regulatory mechanisms and crosstalk.

## Introduction

**Figure 1.**
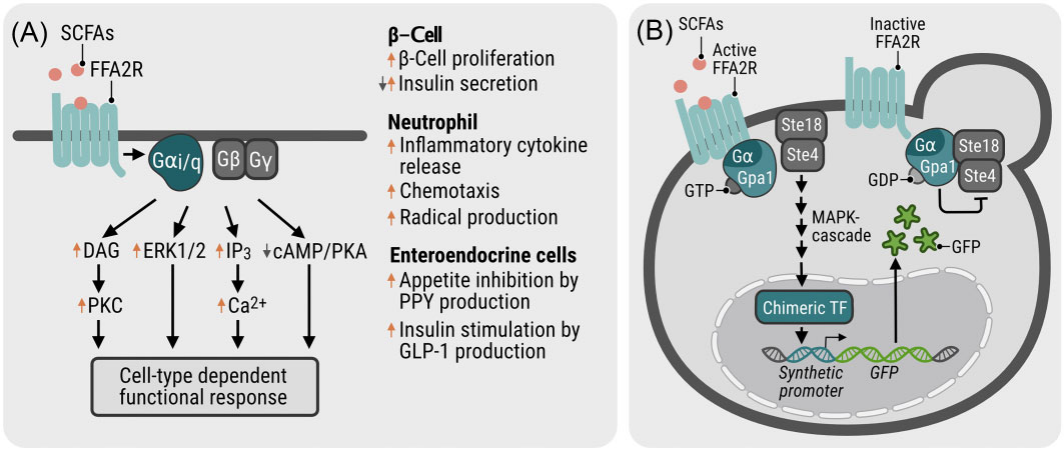
FFA2R-mediated signaling in human cell lines and yeast sensor design. (A) A general overview of the G protein α (Gα)- and G protein βγ (Gβγ)-regulated signaling of FFA2R in human cells in response to receptor activation is presented. FFA2R couples to both the Gα_q/11_ and Gα_i/o_ G protein subunit families, activating an array of cellular signaling pathways and cell-type-specific functional responses, for which examples are listed (Secor et al. 2021, Teyani and Moniri 2023). Note that variations, such as overlap between signaling pathways and crosstalk with other receptors may occur in certain cell types, not included in this general overview. (B) A yeast sensor platform for evaluation of FFA2R was constructed via the yeast mating pathway, with transcription-based fluorescence (GFP) as an output. Upon binding of an agonist to FFA2R, the chimeric Gα (Gpa1-Gα) disassociates from the βγ-heterodimer (Ste18-Ste4) to bind the GPCR. Subsequently, Ste18-Ste4 transmits the signal, activating the MAPK cascade resulting in phosphorylation of the chimeric transcription factor (TF; Ste12-PRD) and transcription of *GFP* under the synthetic promoter *LexO(6x)LEU2p*. To enable rational tuning of the pathway with a predictable response, a parental strain was chosen in which only key mating pathway components remained, making it void of mating pathway crosstalk (Shaw et al. 2019). The expression levels of pathway components depicted in blue and green had been previously optimized for an increased dynamic range of the pathway response. Different chimeric Gα, containing the C-terminus of human Gα_i1_, Gα_i3_, Gα_o_, Gα_q_, and Gα_11_, were evaluated. In addition, expression levels of the Gpa1-Gα _i1_ were further tuned to adjust baseline activity of the pathway. SCFA, short-chain fatty acids; FFA2R, free fatty acid receptor 2; DAG, diacylglycerol; PKC, protein kinase C; IP3, inositol trisphosphate; ERK1/2, extracellular signal-regulated kinase-1/2; cAMP, cyclic adenosine monophosphate; PKA, protein kinase A; MAPK, mitogen-activated protein kinase; GFP, green fluorescent protein; GDP, guanosine diphosphate; GTP, guanosine triphosphate; PPY, pancreatic polypeptide; GLP-1, glucagon-like peptide 1.

The over 800 G protein-coupled receptors (GPCRs) encoded in the human genome play vital roles in virtually all cells and tissues. Binding to a wide array of agonists, including odorants, hormones, and metabolites (Lv et al. 2016), GPCRs are prominent therapeutic targets, making up ∼34% of FDA-approved drug targets as of 2017 (Hauser et al. 2017). Some members of this receptor family are of major importance in modulation of metabolism and immune responses, achieved through activation of biochemical cascades in response to extracellular signals recognized by receptors expressed in cell plasma membranes (Lv et al. 2016, Chen and Obal 2023). A human GPCR of interest is the free fatty acid 2 receptor (FFA2R/FFAR2/FFA2/GPR43). Recognizing short-chain fatty acids (SCFAs) such as acetate, propionate, and butyrate, FFA2R induces a response in several immune cells, the adipose tissue, enteroendocrine cells, and pancreatic β-cells, demonstrating that functional FFA2Rs are expressed in these cells and tissues (Namour et al. 2016, Sergeev et al. 2017, Chun et al. 2019, Secor et al. 2021). FFA2R regulates and modulates a multitude of cellular processes, including β-cell proliferation, insulin secretion, and neutrophil activation, and has been suggested as a promising therapeutic target for management of the inflammatory response and type 2 diabetes (T2D; Fig. 1A; Namour et al. 2016, Bartoszek et al. 2020, Yao et al. 2022, Teyani and Moniri 2023). However, much is still unknown regarding the complex signaling mechanisms of FFA2R with this being an active field of study (Lind et al. 2021, 2023). Research in human cells is further complicated by crosstalk between FFA2R and other receptor-induced signaling pathways (Lind et al. 2023, Teyani and Moniri 2023), such as transactivation (Lind et al. 2023) and coupling between FFA2R and other GPCRs (Ang et al. 2018). While many ligands selectively targeting FFA2R have been developed, so far only one (the antagonist GLPG0974) has reached a Phase II clinical trial for treatment of ulcerative colitis, where it failed to show a clinical improvement within 4 weeks (Pizzonero et al. 2014, Namour et al. 2016, Milligan et al. 2017).

The activating mechanisms of GPCRs are highly conserved among eukaryotes. Specifically, the binding of an agonist to the receptor at the cell surface triggers a structural change that enables coupling to a heterotrimeric GTP-binding protein (G protein) complex located on the cytosolic side of the receptor-expressing cell membrane (Dohlman et al. 1998). The α subunit of the G protein complex, consisting of G protein subunits αβγ (Gαβγ), is bound to GDP in its inactive state. Upon coupling to the activated GPCR, GDP is exchanged for GTP, and the Gα subunit disassociates from the Gβγ-heterodimer (Fig. 1B; Wettschureck and Offermanns 2005). Subsequently, the separated Gα and Gβγ subunits trigger diverse signaling cascades inside the cell, resulting in simultaneous up- and down-regulation of cellular processes (Teyani and Moniri 2023). Although the mechanisms of G protein-mediated signaling are highly conserved, the complexity of signaling varies between species and kingdoms. The human genome encodes 17 different Gα subunit variants divided into 4 families, which are denoted Gα_s_, Gα_i/o_, Gα_q/11_, and Gα_12/13_, based on similarities in expression pattern and protein structure (Wettschureck and Offermanns 2005). In addition, 5 Gβ and 12 Gγ subunit variants are encoded, resulting in a multitude of possible G protein heterotrimers (Wettschureck and Offermanns 2005). The recent analysis of a receptor-G protein *couplome*, obtained from several studies in which the interacting proteins have been overexpressed, revealed that some receptors selectively couple to only one or a few G protein complexes, whereas others are promiscuous and couple to several different ones (Hauser et al. 2022). While it is possible to investigate the G protein recruitment profile for a given GPCR upon overexpressing the GPCR and G proteins, it is difficult to investigate this in primary cells (Wettschureck and Offermanns 2005). This is largely due to these interactions varying not only depending on the cell type, but also depending on the ligand, as the recruitment profile can be partial to specific Gαs (Wettschureck and Offermanns 2005). Examples include the dopamine D_1_ receptor coupling to Gα_s_ and Gα_olf_, respectively, in distinct parts of the brain (Yano et al. 2018) and, relevant for this study, FFA2R coupling to the Gα_i/o_ and Gα_q/11_ subunits in several types of immune cells, including neutrophils, dendritic cells, and monocytes (Schlatterer et al. 2021), and in pancreatic β-cells (Teyani and Moniri 2023). Depending on the cell type, different functions are modulated by the same GPCR (Fig. 1A; Schlatterer et al. 2021, Teyani and Moniri 2023). Further considering the multitude of GPCRs in human cells and crosstalk between these, the process of discerning the response of a specific GPCR to a given stimulus can be difficult and time-consuming. To enable investigation of the receptors in an independent system, yeast-based platforms have been developed for evaluation of human GPCRs (Brown et al. 2015, Wang et al. 2021).

In contrast to primary human cells, baker’s yeast, *Saccharomyces cerevisiae*, only has three GPCRs and two Gα subunit variants, which modulate the mating response and extracellular glucose, with both pathways having been studied extensively (Versele et al. 2001, Harashima and Heitman 2005). Two of the yeast GPCRs (Ste2/Ste3) are involved in the mating response of the respective yeast mating types (a/α), activated by the opposite mating peptide (Versele et al. 2001). Activation results in signal transmission via the corresponding Gα (Gpa1), resulting in cell cycle arrest and transcription of the mating response genes (Versele et al. 2001). The third yeast GPCR (Gpr1) is involved in the glucose response, transmitting the signal via a separate Gα (Gpa2), to regulate pseudohyphal growth in glucose-limited conditions and fermentative growth in the presence of glucose (Kraakman et al. 1999, Versele et al. 2001). Of the two GPCR-regulated pathways, the mating response is less intertwined with the central metabolism of the cell, making it easier to study. As such, the yeast mating pathway has been utilized extensively for diverse applications; as a model for GPCR function, as a platform for screening of ligands of orphan GPCRs, and in recent years as a platform for development of cheap point-of-use biosensors (Versele et al. 2001, Brown et al. 2003, Ostrov et al. 2017, Yasi et al. 2019, Miettinen et al. 2022).

Traditionally, yeast biosensors based on the mating pathway have applied the native pathway with few modifications. These largely include removal of feedback regulation by deletion of genes involved in GPCR desensitization (*SST2*) and α-pheromone degradation (*BAR1*), deletion of genes regulating mating-induced cell-cycle arrest (*FAR1*), and deletion of genes to be replaced, including the mating GPCR gene (*STE2*; Brown et al. 2003, Ostrov et al. 2017, Yasi et al. 2019). When applicable, Gpa1 has also been replaced, introducing a chimeric variant with five C-terminal amino acids of a human Gα, to enable coupling with a larger repertoire of human GPCRs (Brown et al. 2000). For integration of a new GPCR in the strain, a strong constitutive promoter has often been used, while the reintroduced Gα often has been expressed under the native promoter (Brown et al. 2003, Ostrov et al. 2017, Yasi et al. 2019). To enable quantitative measurements of pathway activation, fluorescence-, colorimetry-, or growth-based outputs are commonly utilized (Lengger and Jensen 2019). These are coupled to pathway activation by placing the genes under control of the mating response gene *FUS1* or *FIG1*, both regulated by the transcription factor Ste12 downstream of the mating pathway (Brown et al. 2003, Ostrov et al. 2017, Yasi et al. 2019). While this system has enabled GPCR deorphanization, ligand screening, and screening of mutants (Bindels et al. 2013, Brown et al. 2015, Meltzer et al. 2022), development of fine-tuned yeast sensor strains without a leaky response has been limited by an absence of viable options for pathway adjustment. However, fundamental advances have been made in recent years towards streamlining and modularization of the yeast mating pathway, enabling rational tuning of cell sensing (Shaw et al. 2019). In a heavily engineered strain background void of all nonessential components and connections to the mating response, expression levels of the GPCR, Gα, and output genes have been demonstrated to have a major effect on the pathway response (Shaw et al. 2019). While high expression levels of the GPCR and output genes result in an increased dynamic range and higher sensitivity, the expression level of the Gα regulates a trade-off between an increased basal activity at low expression levels and a decreased dynamic range at high expression levels. In addition, several chimeric Ste12 transcription factors and compatible synthetic promoters were developed, enabling precise adjustment of the output genes expression levels and decoupling from the mating response (Shaw et al. 2019). As such, rational tuning of GPCR-based sensors in yeast has become possible and can potentially aid in improving existing platforms for evaluation of human GPCRs.

In this work, we develop a yeast-based sensor for evaluation of FFA2R ligands acting through Gα_i1_ for application in screening of ligands. Utilizing a strain background with a streamlined mating pathway and increased expression levels of the fluorescent output gene and the GPCR, we aimed to improve the sensitivity and decrease the assay time compared to a previously developed yeast FFA2R sensor (Brown et al. 2003). To optimize the baseline activity of the pathway, varied expression levels of the chimeric Gpa1-Gα_i1_ were evaluated. The yeast platform was verified using SFCAs acetate and propionate, and then tested by evaluation of Gα_i1_-dependent activity of FFA2R towards orthosteric agonists, orthosteric antagonists, and allosteric agonists not previously evaluated in yeast.

## Materials and methods

### Chemicals

Orthosteric agonist 3-benzyl-4-(cyclopropyl-(4-(2,5-dichlorophenyl)thiazol-2-yl)amino)-4-oxobutanoic acid (Cmp1), antagonist ((S)-3-(2-(3-chlorophenyl)acetamido)-4-(4-(trifluoromethyl)phenyl) butanoic acid (CATPB), antagonist 4-[[1-(benzo[b]thiophene-3-carbonyl)-2-methylazetidine-2-carbonyl]-(3-chlorobenzyl)amino]butyric acid (GLPG0974), allosteric agonist ((S)-2-(4-chlorophenyl)-3,3-dimethyl-N-(5-phenylthiazol-2-yl)butanamide (Cmp58), allosteric agonist 4-fluoro-N-[3-[2-[(aminoiminomethyl)amino]-4-methyl-5-thiazolyl]phenyl]benzamide (AZ1729) were kindly provided by the Phagocyte Research group at the Department of Rheumatology and Inflammation Research, University of Gothenburg, Sweden. All listed ligands were dissolved in DMSO to stock concentrations of 10 µM. Subsequent dilutions of the ligands were made in DMSO, to 1000× the concentrations used for induction evaluation.

### Bacterial strains and growth media

*Escherichia coli* DH5α was used for propagation and assembly of plasmids. Cells were cultivated in LB medium (10 g·l^−1^peptone from casein, 10 g·l^−1^ NaCl, 5 g·l^−1^ yeast extract) with addition of the appropriate antibiotic selection (50 mg·l^−1^ kanamycin, 200 mg·l^−1^ ampicillin, or 25 mg·l^−1^ chloramphenicol) after autoclavation. For growth on agar plates, 16 g·l^−1^ agar was added to the media. For cultivation in liquid medium, cells were incubated at 37°C at 180 rpm.

### Yeast growth conditions

For transformation, yeast strains were cultivated in YPD medium [20 g·l^−1^ D(+)-glucose (Merck), 10 g·l^−1^ yeast extract (Merck), 20 g·l^−1^ peptone from meat (Merck)], to which 20 g·l^−1^ agar (Merck) was added to make plates. For selective growth on plates, G418 (200 g·l^−1^) was added to the media.

All media for analysis of strain induction were made 2× concentrated and diluted with sterile MilliQ water upon preparation of the cultures. For precultures before induction, synthetic defined medium (CSM) pH 5.8 [1× CSM: 20 g·l^−1^ glucose, 6.9 g·l^−1^ yeast nitrogen base without amino acids (Formedium), and 0.77 g·l^−1^ CSM (MP Biomedicals)] was used. For evaluation of induction, citrate-phosphate buffered CSM medium with ammonium sulfate and urea pH 6.8 was used (1x CSM-AS/urea: 20 g·l^−1^ glucose, 3.45 g·l^−1^ yeast nitrogen base without amino acids and ammonium sulfate (Formedium), 0.85 g·l^−1^ yeast nitrogen base without amino acids and with ammonium sulfate (Formedium), 2.5 g·l^−1^ urea, and 0.77 g·l^−1^ CSM (MP Biomedicals), 2.35 g·l^−1^ Na_2_HPO_4_, 0.34 g·l^−1^ citric acid) (Prins and Billerbeck 2021). In the case of the ligands acetate and propionate, these were added during media preparation to enable adjustment of pH when needed (1× CSM-AS/Urea-propionate/acetate: 1× CSM-AS/Urea, 10 mM propionate or 10 mM acetate). Other ligands, including AZ1729, Cmp1, Cmp58, CATPB, and GLPG0974, were added to media from 1000× stocks at the time of induction. The media were filter sterilized, also resulting in removal of precipitate due to the high pH. Unless otherwise stated, cells were incubated at 30°C at 225 rpm for growth in liquid culture.

### Yeast strain and plasmid construction

The FFA2R amino acid sequence was retrieved from UniProt (UniProt ID: O15552) and the corresponding DNA sequence was codon optimized for *S. cerevisiae*. To enable pYTK (Lee et al. 2015) and Easy-Clone MarkerFree (Brown et al. 2015) toolkit-based cloning, restriction sites Esp3I, Eco31I, and NotI were excluded from the sequence, and *FFA2R* was ordered with toolkit-compatible overhangs from IDT. Chimeric *GPA1* variants were constructed through truncation of the 5 C-terminal amino acids of Gpa1 and introduction of the 5 C-terminal amino acids of human Gα_i1/i3/o_, replacing the corresponding nucleotides via PCR (Brown et al. 2000) through annealing of oligos containing these and cloning into the pYTK backbone. Plasmids were assembled for integration of the *pCCW12-FFA2R-SSA1t* cassette at site XII-2, *pPGK*-*GPA1-G*α*-tENO2* at XI-5, and *LexO6xLEU2p-HIS3-tENO1-TEF2p-miRFP670-tADH1* at the XI-2 integration site. Another integration plasmid, containing *TEF1p-miRFP670-tCYC1* targeting the XII-1 locus, was received from a colleague (Scott et al. 2022). All plasmids and parts included in assembly are listed in Table S1, oligonucleotides (containing the gRNAs) in Table S2, and expression cassettes and gBlocks in Table S3.

*Saccharomyces cerevisiae* strains in this study were derived from strain Design 4 with a streamlined and optimized mating pathway (Shaw et al. 2019), applying the LiAC/PEG method for transformations (Gietz and Woods 2006). A list of all strains and specifications can be found in Table S4. Seamless CRISPR/Cas9 integrations and deletions were applied to remove the yeast *GPA1, STE2*, and *URA3* genes and to integrate chimeric *GPA1-G*α*_i1/i3/o_* and *FFA2R* into the genome. Cas9 and gRNA were expressed from the same plasmid, using the Cas9-KanMX plasmid backbone with a gRNA expression cassette (Hedin et al. 2023). The gRNA expression cassette was amplified in two parts from the Cas9-KanMX plasmid with the gRNA, separating the upstream and downstream regions of the gRNA spacer in different fragments. By using primers that contained the new gRNA spacer sequence in the tail, the new gRNA spacer could be introduced. The upstream and downstream fragments, containing the new gRNA spacer, were then assembled into a Pfl23II pre-digested Cas9-KanMX backbone by Gibson assembly.

### Evaluation of growth

The developed biosensor strains bearing the *FFAR2* and different chimeric versions of the *GPA1-G*α*_i1/i3/o/q/11_* genes in the ASC4 strain background were cultured in citrate-phosphate buffered CSM-AS/urea to assess their growth performance. Two control strains, including the minimized pathway strain (Design 4) and a strain developed from ASC4, both with the wild-type yeast *GPA1* and mating GPCR *STE2* genes. Briefly, three independent colonies of strains ASC4G0-1, ASC4G1-3, ASC4G2-3, ASC4G3-3, ASC4G9-3, ASC4G10-3, ASC4G1h1-3, ASC4G1h2-3, ASC4G1h3-3, and Design 4 (Table S4) were spiked into 2 ml of CSM in 12 ml tubes and incubated overnight with constant shaking at 30°C. Each fresh preculture was washed with sterile water and inoculated in technical triplicates in 250 µl of citrate-phosphate buffered CSM-AS/urea to an OD_600_ of 0.05 in a 96-half-deepwell microplate. The plate was incubated in a Growth Profiler 960 (Enzyscreen) at 30°C with 250 rpm shaking over a course of 50 h. Green values were recorded every 30 min and were converted to OD_600_ through a standard curve. OD_600_ values were represented over time (Fig. S1A) and maximum growth rate (μ_max_) was obtained by fitting the OD_600_ dependency on time to a linear model in the initial exponential growth phase. The maximum OD_600_ was extracted at the highest point during the 50-h incubation.

### Transient induction of strains in microplate reader

Strains were induced and incubated in a microplate reader to identify the Gpa1-Gα chimera producing the strongest response. Transient measurements of cell density and GFP fluorescence enabled pinpointing of an appropriate incubation time for further evaluation. Three replicate colonies of strains ASC4G1-3, ASC4G2-3, and ASC4G3-3 were used to inoculate 5 ml CSM in a 50-ml falcon tube and were incubated overnight. Cells were harvested by centrifugation for 5 min at 3000 rcf and resuspended in 1x CSM citrate-phosphate buffered CSM-AS/urea to an OD_600_ of 0.1. Five hundred microliters of the cell suspension was added to a 48-well FlowerPlate® (m2p-labs) and diluted with 500 µl 2× citrate-phosphate buffered CSM-AS/urea with/without 20 mM propionate, and remaining ligands were added from 1000× concentrated stocks. Plates were sealed with a sealing foil for reduced evaporation (m2p-labs) and incubated 30°C and 1200 rpm in a Biolector® (m2p-labs) using the BioLection 2 software. Biomass (scattered light as a proxy of biomass) and GFP (excitation: 488 nm, emission: 520 nm with a gain of 50) values for each sample were collected every 30 min. GFP fluorescence data were normalized by the biomass of the respective replicate before analysis.

### Flow cytometer analysis

The FFA2R-induced GFP signal in response to different concentrations of ligands was analyzed by flow cytometry (Guava easyCyte, Luminex). Three replicate colonies of strain ASC4G1-3 were used to inoculate 5 ml CSM-AS/urea pH 5.8 in 50-ml Falcon tubes and incubated overnight. Cells were harvested by centrifugation for 5 min at 3000 rcf and resuspended to an OD_600_ of 5 in sterile MilliQ water. For induction, cells were diluted tenfold to an OD_600_ of 0.5 in 250 µl CSM-AS/urea pH 6.8 with ligands of interest in a 96-well round-bottom plate (Axygen, *P*-96–450R-C-S). The plate was sealed with a 96-well plate sandwich cover and incubated at 250 rpm at 30°C for 3 h before evaluation. Cells were typically evaluated at 8 different ligand concentrations for regression analysis or four different concentrations for simple evaluation of the response intensity, with 3 biological replicates per condition. For evaluation of the effect of increasing concentrations of a ligand, all conditions and replicates were analyzed on the same 96-well plate. For analysis by flow cytometry, cells were diluted to an OD_600_ of 0.02 in 200 µl sterile MilliQ water in a 96-well round well plate (163320, Thermo Scientific™). Fluorescent proteins were excited with a 488 nm laser (for GFP) or 648 nm laser (for miRFP670), with recorded fluorescence intensity being expressed in arbitrary units. Data were collected for 5000 events per replicate.

### Data analysis

All data analysis was performed in R (version 4.3.1) with the tidyverse, hexbin, ggridges, ggtext, ggpubr, scales, minpack.lm, viridis, ggridges, growthrate, reshape2, and readr packages. All scripts and data are available on GitHub.

For analysis of flow cytometry data, the population of each biological replicate analyzed in parallel during the same experimental run was filtered by forward scatter (FSC), side scatter (SSC), and red fluorescent protein (RFP) intensity (log(FSC)>2.8, log(SSC)<4.8, log(RFP)>0.5). This enabled filtering out non-viable cells, lacking RFP, as well as cells of unusual size or granularity by FSC and SSC, respectively. The mean GFP fluorescence was calculated, followed by normalization to the uninduced control of the same replicate. This enabled calculation of fold change over baseline. Data shown represent the mean and standard deviation of these three replicates. A one-sided *t*-test was applied for pairwise comparison between conditions, and a two-way ANOVA was applied when comparing across multiple factors.

The parameters of a three-parameter sigmoidal curve, following equation (1), were fit to the data using the function nlsLM.


(1) \begin{eqnarray*}
E = {E_{min}} + \frac{{{E_{max}} - {E_{min}}}}{{1 + {{10}^{\left( {pEC50 \,-\, \log \left( x \right)} \right)*n}}}}
\end{eqnarray*}

*E* represents the effect, *E_min_* the effect at baseline, and *E_max_* the maximal effect size; *pEC50* is the log-transformed concentration at 50% of the maximal effect, the variable *x* is the concentration at which E was measured, and *n* is the slope of the curve. By fixing n to 1, a three-parameter regression was performed.

## Results

### Reporter strain construction and initial assessment

In order to investigate Gα-dependent signaling through the yeast mating pathway, a parental strain void of mating pathway crosstalk, Design 4, with a streamlined mating pathway and GFP as a transcriptional output under the *LexO6xLeu2p* promoter output was selected (Shaw et al. 2019). Expression cassettes of yeast G protein α (Gpa1) and mating GPCR (Ste2) were deleted from the genome of this strain to enable reintegration in different genomic loci with select promoters (Otto et al. 2021). By replacing these with *FFA2R* expressed from integration site XII-2 and either of chimeric Gpa1-Gα_i1_, Gpa1-Gα_i3_, Gpa1-Gα_o_, Gpa1-Gα_q_, or Gpa1-Gα_11_ (Brown et al. 2000) expressed from integration site XI-5 (Lee et al. 2015), FFA2R was coupled to the yeast mating pathway. A control strain with the yeast *GPA1* and *STE2* genes integrated in the same sites was constructed as well. To enable similar expression levels of the reintegrated genes, the same set of promoters and terminators was used as in the parental strain for the respective genes. While not utilized in this study, constitutive expression of *miRFP670* was implemented in the strain to enable normalization of the flow cytometry data using RFP as an indicator of cells size and viability. In addition, an inducible histidine expression cassette with *HIS3* expressed from the *LexO6xLeu2p* promoter was integrated together with RFP, to enable the possibility of growth-based selection in future studies, and the *URA3* gene was deleted to enable selection based on prototrophy.

To assess possible effects on growth performance caused by the further modifications of the parental strain, a growth assay with the newly developed strains (Table S4) was conducted, and growth parameters were obtained. Comparing the growth rate (μ_max_) of these strains to that of the parental background strain (Design 4), the newly constructed strains performed in line with the parental control (Fig. S1A and B). Conversely, the newly generated strains reached a higher maximum OD_600_ compared to the parental strain (Fig. S1A and C), with this effect being apparent after the exponential growth phase. As the induction response is evaluated within the exponential phase, the difference in maximum OD_600_ should not affect the evaluation of induction in the developed strains.

**Figure 2.**
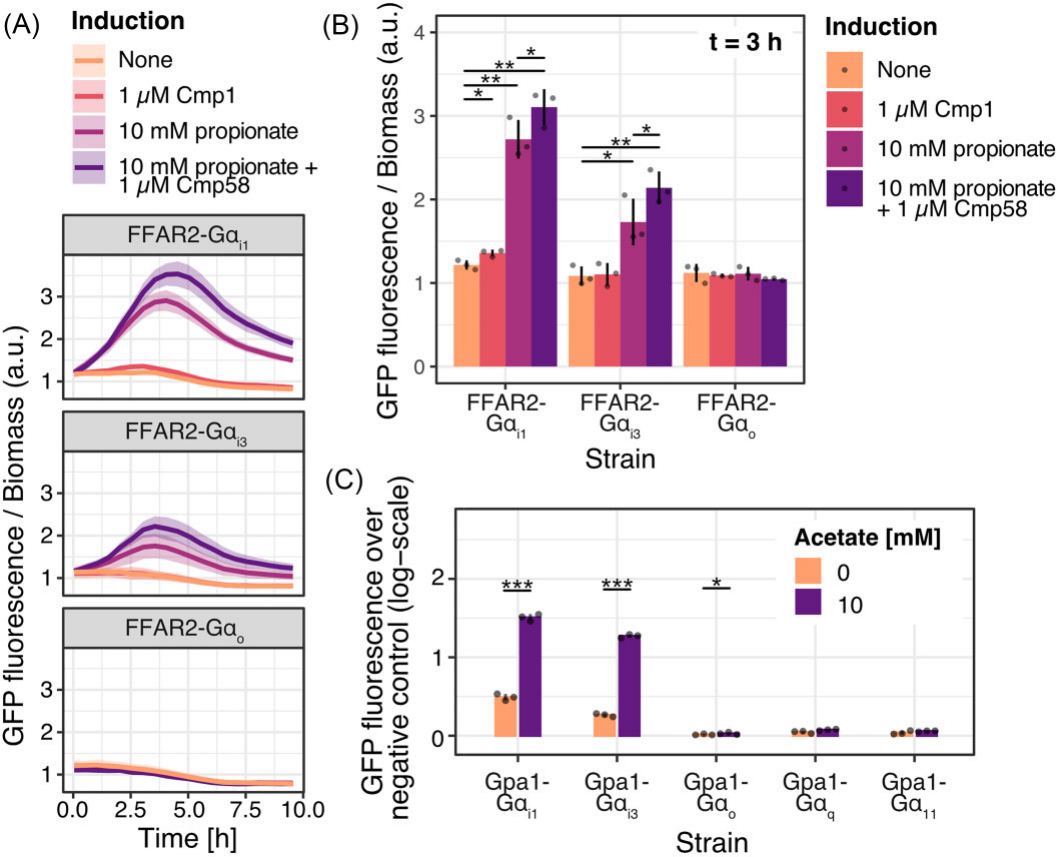
Transient GFP expression induction for different Gpa1-Gα_i/o_ chimeras and evaluation of Gpa1-Gα activity by flow cytometry. Plots (A and B) display the transient GFP fluorescence normalized to cell density measured in a microbioreactor during 10 h following addition of the ligands, and (C) the flow-cytometer-based evaluation of fluorescence over a baseline control. (A) Three strains, expressing Gpa1-Gα_i1_, Gpa1-Gα_i3_, and Gpa1-Gα_o_, were investigated after incubation with ligands of interest (see legend). The response is expressed as the mean (line) GFP fluorescence/biomass, and the shaded area represents the s.d. (*n* = 3). (B) The GFP fluorescence per biomass after 3 h of induction. Bars represent the mean (*n* = 3) and vertical lines the s.d., with dots representing the measurement of the biological strain replicates. (C) The baseline expression over a Gα-negative (*Δgpa1*) control strain was evaluated for acetate induction after 3 h of incubation. Bars represent the mean (*n* = 3) GFP fluorescence over the negative control at the respective induction concentrations and vertical lines represent the s.d. Dots represent the mean fluorescence of 5000 individual cells analyzed by flow cytometry for each biological replicate. Additional strains, Gpa1-Gα_q_ and Gpa1-Gα_11_, were included. *P < 0.05, **P < 0.01, **P < 0.001, one-sided *t*-test.

Next, an appropriate incubation time of cultures after induction was investigated by the transient monitoring of cell-density-corrected fluorescence intensity of induced cultures in a microplate reader (Fig. 2A and B). Three of the constructed strains, with chimeric G proteins belonging to the Gpa1-Gα_i/o_ subgroup were evaluated with three separate sets of inducers. These include natural orthosteric agonist propionate and synthetic orthosteric agonist Cmp1, occupying the primary binding pocket to activate the receptor, and synthetic allosteric agonist Cmp58, binding outside the primary binding pocket to produce additional activation to that produced by orthosteric agonists. The synthetic compounds were originally developed for potential therapeutic applications, with Cmp1 targeting FF2AR with improved potency and selectivity compared to the natural agonists (Hudson et al. 2013) and Cmp58 positively modulating the receptor activation (Wang et al. 2010).

Strains were evaluated over the course of 10 h after addition of inducers, reaching the peak activation relative to the biomass between 3 and 5 h (Fig. 2A). To achieve the shortest possible assay time, 3 h of incubation was selected for further evaluation. At this incubation time, there was a significant increase in cell density-normalized fluorescence intensity in response to propionate in strains with Gpa1-Gα_i1_ and Gpa1-Gα_i3_ compared to when no ligand was added (*P* = 0.003 and *P* = 0.011, one-sided *t*-test; Fig. 2B). In these strains, cells incubated with both propionate and Cmp58 further produced a significant increase in fluorescence compared cells incubated with propionate alone (*P* = 0.030 and *P* = 0.001, one-sided *t*-test; Fig. 2B). The strain with Gpa1-Gα_i1_ further produced a significant response to 1 µM Cmp1 compared to when no ligand was added (*P* = 0.030, one-sided *t*-test; Fig. 2B), while the other strains did not. Interestingly, in the strain with Gpa1-Gα_i3_, the overall intensity of the GFP fluorescence response was weaker, likely contributing to the absence of response to Cmp1. The strain expressing Gpa1-Gα_o_ showed no signs of activation, in accordance with published data (Fig. 2A and B; Brown et al. 2003). With this, 3 h was confirmed to be an appropriate incubation time to be applied in all subsequent analysis.

Having identified an appropriate incubation time, we then sought to evaluate all the constructed strains, including strains with chimeric G proteins from both the Gpa1-Gα_i/o_ and the Gpa1-Gα_q/11_ subgroups, using flow cytometry. This method allows for determination of the fluorescence intensity produced by a yeast cell population in high resolution, while enabling comparison of the cultures by the mean fluorescence intensity produced (Fig. S2). For induction, 10 mM of the natural agonist acetate was used. Furthermore, the baseline expression of the respective strains in the absence of inducer was evaluated by including a negative control strain, lacking a Gpa1-Gα expression cassette, to enable subtraction of the mean background fluorescence at each induction concentration. Out of the five evaluated strains, the only strains with a significant response compared to the uninduced state were those with Gpa1-Gα_i1_, Gpa1-Gα_i3_, and Gpa1-Gα_o_ (*P* < 0.001, *P* < 0.001, and *P* < 0.05, one-sided *t*-test; Fig. 2C). However, the induction in the strain with Gpa1-Gα_o_ was barely noticeable. Several strains had a significant baseline expression of GFP at 0 nM induction when compared to the Gα-negative control, including strains with Gpa1-Gα_i1_, Gpa1-Gα_i3_, Gpa1-Gα_q_, and Gpa1-Gα_11_ (*P* < 0.01, *P* < 0.001, *P* < 0.05, and *P* < 0.05, one-sided *t*-test; Fig. 2C). Interestingly, strains with Gpa1-Gα_q_ and Gpa1-Gα_11_ exhibited a slight baseline expression, although these strains did not get activated by the ligand. Out of the evaluated strains, the strain with Gpa1-Gα_i1_ produced the strongest response but also had the strongest baseline expression. As such, this strain was chosen for further tuning of the yeast mating pathway by varying the level of Gpa1-Gα_i1_ expression, to evaluate if the response intensity could be increased and the baseline expression adjusted.

**Figure 3.**
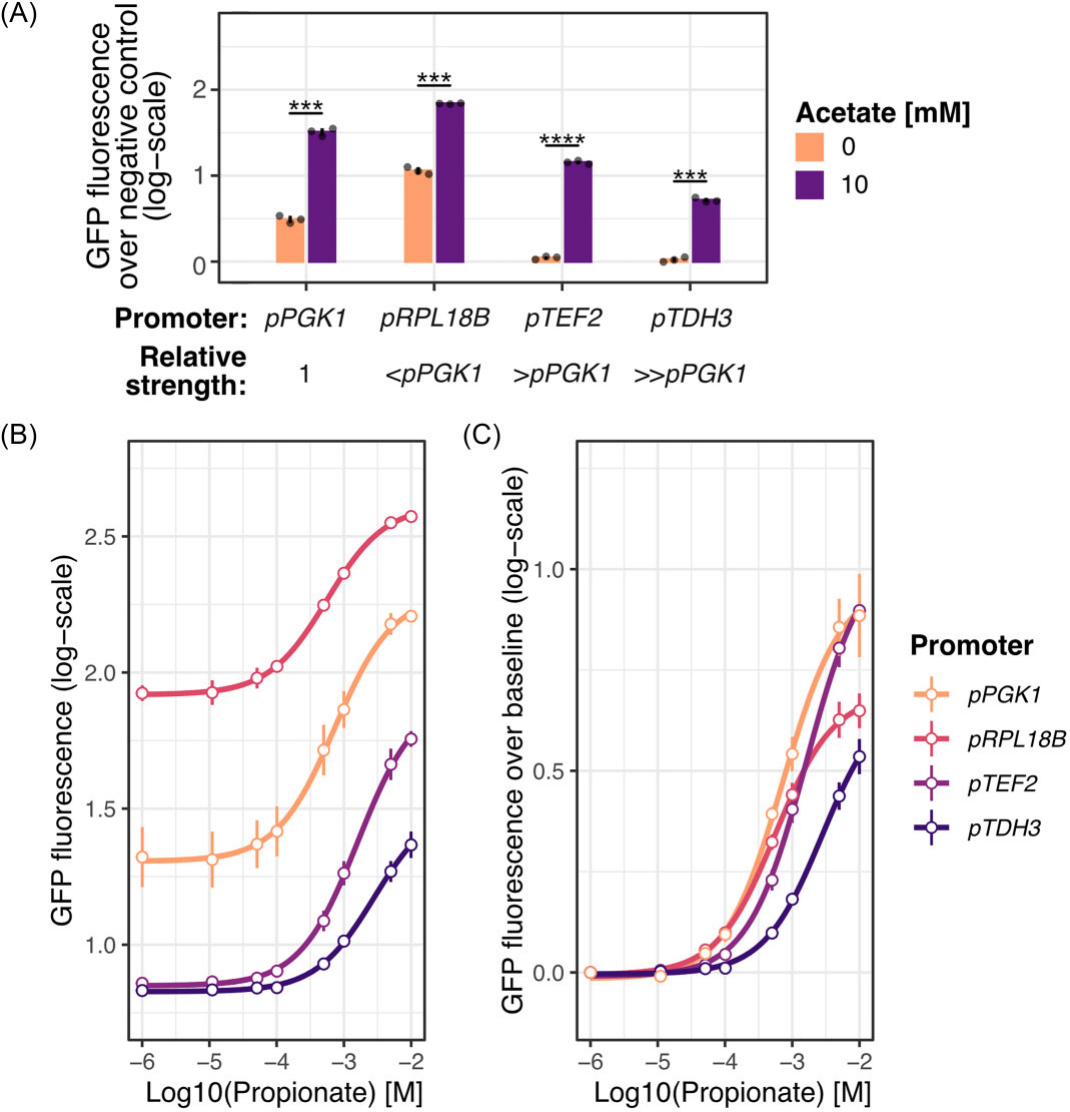
Tuning of Gpa1-Gα_i1_ expression levels. Strains were constructed with Gpa1-Gα_i1_ expressed from promoters stronger or weaker than *pPGK1*, the promoter used in the initial strain evaluation. (A) The bar plot represents baseline expression over the Gα-negative (*Δ gpa1*) control strain, evaluated in strains with varied Gpa1-Gα_i1_ expression levels after 3 h of incubation. Bars represent the mean GFP fluorescence over the negative control for biological replicates and vertical lines represent the s.d. (*n* = 3). Dots represent the mean GFP fluorescence of flow cytometry data for each biological replicate. The presented activation curves represent the log10-transformed GFP fluorescence response (B) and log10-fold change of GFP fluorescence over the baseline, (C) respectively (dots), after 3 h of incubation. Fold-change over baseline was calculated by subtracting the uninduced GFP fluorescence intensity of the respective strain and replicate. Vertical lines represent s.d. (*n* = 3) for all but strain *pPGK1* (*n* = 2), as an outlier replicate was removed. *P < 0.05, **P < 0.01, **P < 0.001, one-sided *t*-test.

Three different constitutive promoters were evaluated for expression of Gpa1-Gα, in addition to the previously applied promoter *pPGK1*. These include the weaker promoter *pRPL18B* and the two stronger promoters *pTEF2* and *pTDH3*, with promoter strength increasing in the given order (Lee et al. 2015). Upon evaluation of the baseline expression of each strain and the response intensity, all strains produced a significant increase in GFP fluorescence upon induction independent of the promoter (Fig. 3A). However, only the three strains with promoters *pPGK1, pRPL18B*, and *pTEF2* produced a significant expression over the baseline control in the absence of inducer (*P* < 0.01, *P* < 0.001, and *P* < 0.05, one-sided *t*-test; Fig. 3A). The baseline fluorescence decreased with increasing promoter strength, and while it was significant for the strain with promoter *pTEF2* compared to the Gα-negative control strain, the baseline GFP fluorescence was barely noticeable. To better understand the effect of the different promoters used for Gpa1-Gα_i1_ expression, the activation of strains with increasing concentrations of propionate was evaluated (Fig. 3B and C). The activation curves are presented both as raw GFP fluorescence intensity measurements (Fig. 3B) and as baseline-corrected curves (Fig. 3C), where the GFP fluorescence intensity in the uninduced state for each strain and replicate is subtracted at each induction level. As the data are log transformed, the latter corresponds to fold-change over baseline.

**Figure 4.**
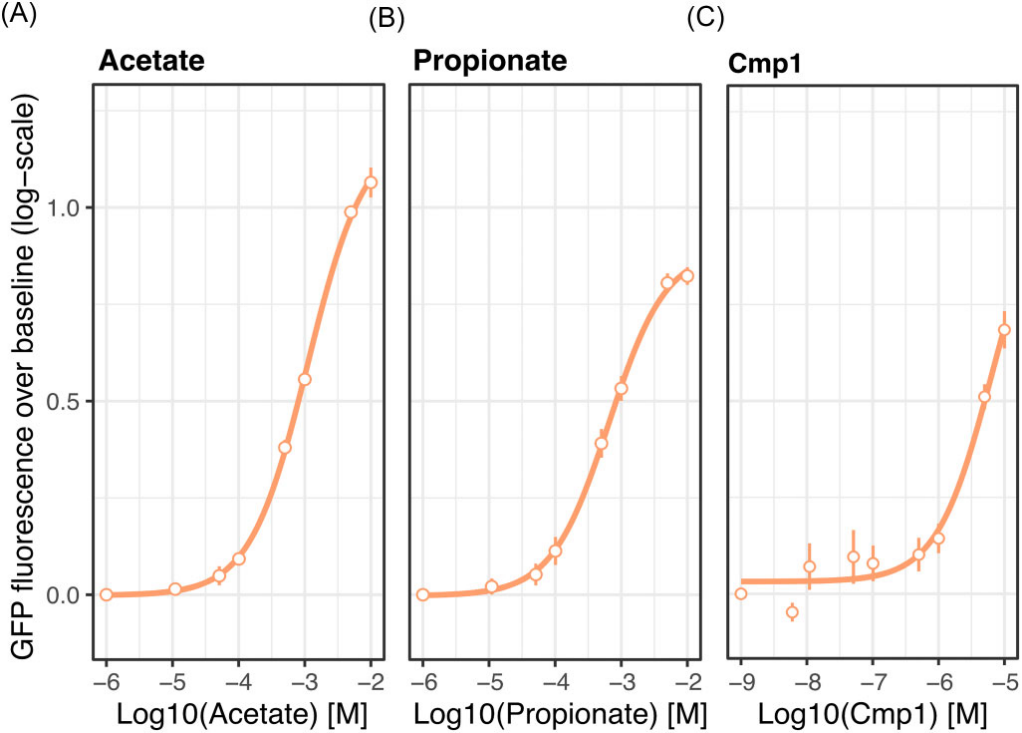
Propionate, acetate, and the synthetic orthosteric agonist Cmp1 strongly induce Gα_i1_-dependent signaling. The effect of varying concentrations of (A) acetate, (B) propionate, and (C) Cmp1 on GFP expression induction in yeast with Gpa1-Gα_i1_-dependent signaling after 3 h of incubation. The response is expressed as mean log10-fold change of GFP fluorescence over the baseline (dots), calculated from the mean of flow cytometry data; vertical lines represent s.d. (*n* = 3).

Investigating the activation curves of the different strains, the difference in baseline GFP fluorescence intensity is clear throughout the range of evaluated concentrations (Fig. 3B). Interestingly, the strains with the strongest and weakest promoters, *pTDH3* and *pRPL18B*, had a decreased dynamic range, while the dynamic range of strains with promoters *pPGK1* and *pTEF2* were similar (Fig. 3B and C). Strains with promoters *pRPL18B, pPGK1*, and *pTEF2* were confirmed to have a limit of detection (LOD) of 0.05 mM propionate (*P* < 0.01, *P* < 0.05, and *P* < 0.05, one-sided *t*-test), while the strain with promoter *pTDH3* had a LOD of 0.1 mM (*P* < 0.05, one-sided *t*-test; Fig. 3C). Due to one of the replicates of the strain with *pPGK1* being an outlier, the LOD of this strain was evaluated in a separate experimental run (Fig. 4A). Overall, we found that in terms of both dynamic range and sensitivity, strains with the *pTEF2* and *pPGK1* promoters performed similarly, with the former having close to no baseline GFP fluorescence activity and the latter having a high baseline activity. For the purpose of screening both agonists, producing pathway activation upon binding, and inverse agonists, resulting in suppression of the pathway signaling upon binding, a baseline expression centered between the background (no GFP fluorescence) and GFP fluorescence saturation is beneficial, allowing equal opportunity for activation and suppression. For this purpose, the strain with promoter *pPGK1* was selected for all further evaluation of ligands acting through Gα_i1_-mediated signaling, after confirming that incubation with an orthosteric antagonist resulted in suppression of the signal (Fig. S2). Although this strain appears to have a higher dynamic range for activation compared to suppression, it had the closest to the ideal ratio out of the evaluated strains, while maintaining a high sensitivity.

### Activation of FFA2R by orthosteric agonists

To characterize the strain for evaluation of Gα_i1_-mediated FFA2R activation, the dose-dependent response to the natural SCFAs agonists propionate and acetate was investigated. For both acetate and propionate, the average GFP fluorescence in cells increased in a concentration-dependent manner, with similar estimates for pEC50 (pEC50_Ac_ = 2.96 ± 0.02 and pEC50_Pr_ = 3.18 ± 0.03, curve upper asymptote was not accurately defined; Fig. 4A and B). However, comparing the dynamic range and relative response intensity at higher induction concentrations, acetate appears to be the more potent inducer. The LOD was identified as 0.05 mM (*P* < 0.05, one-sided *t*-test) and 0.05 mM (*P* < 0.05, one-sided *t*-test) for acetate and propionate, respectively. As such, the sensor displayed similar sensitivity for acetate and propionate, while acetate produced a higher intensity response through the mating pathway via Gpa1-Gα_i1_-specific coupling to FFA2R compared to propionate.

Next, the dose-dependent response to the synthetic orthosteric agonist Cmp1 was evaluated, a ligand with a 3-fold higher potency compared to propionate based on pEC50 values in human cell lines (Schmidt et al. 2011, Hudson et al. 2013). In our platform, the estimated value for Cmp1 (pEC50_Cmp1_ = 5.14 ± 0.17, upper asymptote not defined; Fig. 4C) was 2.18-fold higher than for acetate and 1.96-fold higher than for propionate. The LOD of Cmp1 was identified as 0.1 µM (*P* < 0.05, one-sided t-test), at which a significant increase in response was produced compared to in the absence of inducer. With this, it was confirmed that the two tested SCFAs and Cmp1 produced a dose-dependent response in the yeast platform. In addition, the Gα_i1_-dependent activation of Cmp1 was confirmed in a yeast platform for the first time.

### Effect of antagonists on the yeast FFA2R platform

**Figure 5.**
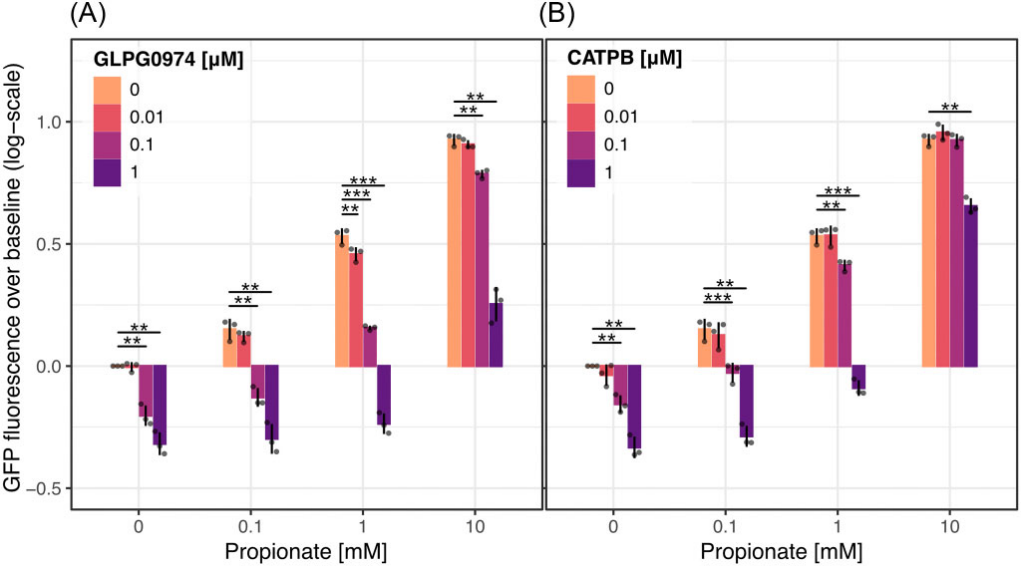
Antagonists GLPG0974 and CATPB repress GFP expression in absence and presence of propionate. The effect of varying concentrations of (A) propionate with GLPG0974 and (B) propionate with CATPB on GFP expression induction in yeast with FFA2R-Gpa1(Gα_i1_) after 3 h of incubation. Bars represent the mean log10-fold change of GFP fluorescence over the baseline of three replicates, shown as dots, and significance is calculated compared to the baseline (*n* = 3). **P < 0.01, ***P < 0.001, one-sided *t*-test.

Next, the suppressive effect of synthetic antagonists GLPG0974 and CATPB, originally developed for potential therapeutic applications (Park et al. 2016, Milligan et al. 2017), was investigated in the presence of increasing propionate concentrations. Both antagonists are known to be orthosteric, repressing activation in concentration-dependent manner in stable human cell lines with overexpression of FFA2R (Sergeev et al. 2016, 2017). Upon evaluation of the dose-dependent suppression of pathway activation in yeast, GLPG0974 and CATPB were both found to decrease the GFP fluorescence intensity in a concentration-dependent manner. Both compounds produced significant suppression at 0.1 µM in both the absence and presence of propionate compared to in the absence of the antagonists (*P* < 0.01 and *P* < 0.01, one-sided *t*-test), resulting in a decreased GFP fluorescence intensity below baseline in the absence of propionate (Fig. 5A and B). Comparing the two antagonists in terms of potency, the relative signal suppression resulting from GLPG0974 (Fig. 5A) was stronger than that of CATPB (Fig. 5B). With this, concentration-dependent signal suppression by both compounds in the presence and absence of an orthosteric agonist was demonstrated on this platform, as well as an inverse agonist behavior of both GLPG0974 and CATPB.

### Evaluation of allosteric modulators

**Figure 6.**
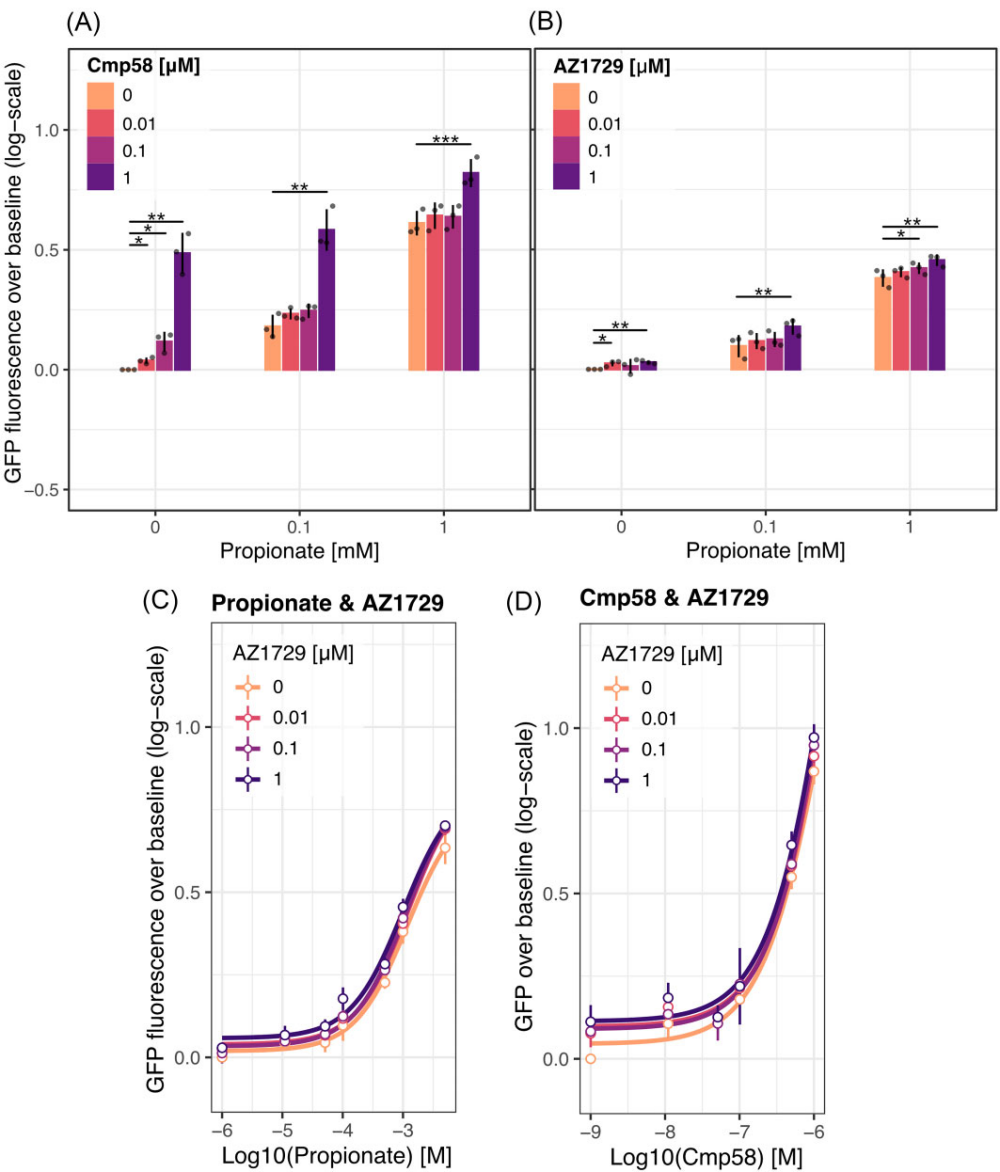
Allosteric agonists Cmp58 and AZ1729 exhibit an agonistic response in the yeast platform. Bar plots display the effect of varying concentrations of (A) propionate and Cmp58 and (B) propionate and AZ1729 after 3 h of incubation. Bars represent the mean log10-fold change of GFP fluorescence over the baseline of three replicates, shown as dots, and significance is calculated compared to the baseline (*n* = 3). The effect of varying concentrations of (C) propionate with AZ1729 and (D) Cmp58 with AZ1729 on GFP expression induction in yeast with FFA2R-Gpa1(Gα_i1_) after 3 h of incubation. The response is expressed as mean log10-fold change of GFP fluorescence over the baseline (dots), calculated from the mean of flow cytometry data, and vertical lines represent s.d. (*n* = 3). *P < 0.05, **P < 0.01, ***P < 0.001, one-sided *t*-test.

Lastly, the effect of the allosteric agonists/modulators Cmp58 and AZ1729 was evaluated, both known to positively modulate the response of the receptor in the presence of agonists (Wang et al. 2010, Bolognini et al. 2016). Relative to propionate, Cmp58 reportedly has a 2.7-fold higher pEC50 and AZ1729 a 2.9-fold higher pEC50 in human cell lines with constitutive FFA2R expression, while these vary depending on which pathway output is measured (Fig. 1A; Wang et al. 2010, Schmidt et al. 2011, Bolognini et al. 2016). In the yeast sensor platform, both compounds acted as allosteric agonists through the Gα_i1_ subunit, producing a concentration-dependent upshift in GFP fluorescence intensity in both the presence and absence of propionate (Fig. 6A and B). With increasing propionate concentration, the activating effect of Cmp58 decreased as a result of signal saturation (Fig. 6A). In addition, AZ1729 was found to slightly sensitize FFA2R towards propionate in the lower propionate concentration range (0–0.1 µM, *P* < 0.05, ANOVA), producing an amplification in signal compared to the individual responses of AZ1729 and propionate taken together (Fig. 6C). However, this effect was weak and absent at higher concentrations of propionate (Fig. 6C). Of the two allosteric agonists, Cmp58 produced a stronger signal, comparable with that of the orthosteric agonist Cmp1 in absence of propionate (Fig. 6A and D), while the activation intensity of AZ1729 at evaluated concentration was weaker albeit significant (Fig. 6B). Both compounds were found to have an LOD of 0.01 µM in absence of propionate (*P* < 0.05, one-sided *t*-test), while 1 µM AZ1729 and Cmp58 respectively produced a significant increase in GFP fluorescence throughout increasing propionate concentrations compared to in the absence of the allosteric modulators (*P* < 0.01, one-sided *t*-test; Fig. 6A and B). These results confirm the allosteric agonist activity of AZ1729 and Cmp58 via Gα_i1_, while neither compound acted as an allosteric modulator to produce cooperative effects together with propionate.

As a final evaluation, simultaneous stimulation by AZ1729 and Cmp58 was investigated, as these have been shown to bind separate sites in the receptor and produce cooperative activation in human neutrophils (Lind et al. 2020). Again, both compounds did produce a concentration-dependent increase in GFP fluorescence, indicating separate binding sites in FFA2R (Fig. 6D). However, no cooperative effects were found (Fig. 6D).

## Discussion

Here, we present a yeast platform for the evaluation of Gα_i_-mediated signaling by human FFA2R, constructed in a yeast strain background with a streamlined mating pathway void of nonessential components. By altering the Gα expression levels, baseline output levels were tuned. Utilizing GFP as a fluorescent output, the response time of this sensor is shorter than that reported for previously developed FFA2R sensors, with an assay time of 3 h after incubation as compared to 24 h (Brown et al. 2003, 2015), making induction assays increasingly efficient. While other Gα variants known to interact with FFA2R in human cells were expressed under the same promoter, the only Gpa1-Gα producing a significant response besides Gα_i1_ was Gα_i3_. Out of the two, both had similar dynamic ranges, with the baseline of Gα_i1_ being higher. This is in accordance with previously reported results for FFA2R yeast sensors (Brown et al. 2003). We did, however, find that both Gα_q_ and Gα_11_ produced a slight but significant baseline activation, which indicates there may be some interaction between the GPCR and chimeric Gα. Due to the signal being weak, however, this was not further pursued. Out of the functional chimeric Gα, Gα_i1_ was chosen for further tuning to enable comparison to previously published yeast FFA2R sensors in the literature.

In our case, the modularity of the streamlined mating pathway enabled evaluation of varied expression levels of Gpa1-Gα_i1_, allowing for tuning of the activation response curve in terms of sensitivity, dynamic range, and baseline expression (Fig. 3A and B). While two of the evaluated promoters, *pTEF2* and *pPGK1*, produced equally high sensitivity and dynamic range for sensing of propionate, the difference in baseline expression makes these applicable in different screening scenarios. With the higher Gα expression level under promoter *pTEF2*, the baseline expression was reduced to a barely detectable level. This would be suitable for applications where a clear distinction between an active and inactive sensor is beneficial, such as in diagnostics or in screening of mutant GPCR libraries. However, for applications such as ligand screening, baseline activity is key for detection of the full range of ligands, as it allows for detection of inverse agonists. As such, promoter *pPGK1* was selected for Gα expression in further strain evaluations, due to the baseline expression of this strain allowing for bidirectional detection of ligands. In contrast, strains expressing Gα from the *pTDH3* and *PRPL18B* promoters suffered either a decrease in sensitivity due to an abundance of the Gα causing inhibition of the Gβγ complex, or a decreased dynamic range due to a lack of Gα resulting in signal saturation at lower inducer concentrations. Interestingly, the developed strains did not present an increased sensitivity to the SCFAs compared to the previously developed yeast sensors (Brown et al. 2015), as was expected based on the increased expression levels of the GPCR and the output gene in the background strain (Shaw et al. 2019). Based on this, it is possible that a limited interaction between the GPCR and the chimeric Gα in the yeast strain is preventing an enhanced response.

As acetate is part of the central yeast metabolism, there is a possibility that the observed baseline activity could be caused by the small amounts of acetate produced by *S. cerevisiae* (Leupold et al. 2019). While this possibility cannot be excluded, yeast sensors based on the mating pathway can produce baseline activity in the absence of a ligand if the relative expression levels of key pathway genes are not optimized (Shaw et al. 2019). However, to guard against this being the case, one could opt for optimization of cultivation conditions or engineering of the strain background for decreased acetate production. Considering the results presented in this work, however, it is unlikely that this would have major effects on the application towards identification of novel ligands, while it should be taken into account during the interpretation of the ligands´ mode of action.

With several modifications having been introduced into the already heavily engineered parental strain, Design 4, the growth of strains was evaluated to determine the effects of these. This revealed a major difference in growth between the parental strain and the newly constructed ones, largely residing in the low maximum OD_600_ reached by the parental strain. Interestingly, this strain showed no aerobic growth phase, instead displaying a drop in cell density after the exponential phase. As such, it appears that while the newly constructed strains have additional expression cassettes integrated, the relocation of expression cassettes for the GPCR and Gα from the *URA3* to new integration loci resulted in a positive effect on growth. The observed behavior could be related to an excessive metabolic burden being relieved, as gene expression efficiencies can vary greatly depending on their integration loci (Bai Flagfeldt et al. 2009). Even so, the strains performed similarly in the exponential phase and produced no significant difference in growth rate. As such, the observed differences in the anaerobic phase are unlikely to affect ligand detection within the assay time of 3 h.

To validate the sensor strain, activation by both orthosteric and allosteric agonists, as well as suppression by orthosteric inverse agonists, was characterized. Upon evaluation of activation by SCFAs acetate and propionate (Fig. 5A and B), the dose-dependent activation was found to be in accordance with the literature (Schmidt et al. 2011, Hudson et al. 2013). Both SCFAs reportedly have a similar potency in human cell lines, with the stronger agonist varying depending on the measured output (Schmidt et al. 2011). In stable human cell lines with both Gα_i/o_ and Gα_q/11_ subunit families, acetate showed a higher potency in G protein activation assays (pEC50_Ac_ = 4.43 and pEC50_Pr_ = 3.99), while propionate resulted in increased accumulation of the downstream metabolite IP3 (pEC50_Ac_ = 3.94 and pEC50_Pr_ = 4.66; Fig. 1; Schmidt et al. 2011). In comparison, the estimated pEC50s for acetate and propionate in the presented yeast platform are somewhat lower (pEC50_Ac_ = 2.96 ± 0.02 and pEC50_Pr_ = 3.18 ± 0.03) with propionate having a slightly lower pEC50 compared to acetate. Instead looking into the apparent LOD in the same stable human cell line, initial activation seems to be detectable around 0.01 mM for both acetate and propionate (Schmidt et al. 2011, Hudson et al. 2013), which is slightly lower than the LOD of 0.05 mM for both acetate and propionate identified in our strain. Conversely, the maximum activation of the developed sensor strain was not yet reached at 10 mM acetate, while the saturation was reached at lower concentrations in the stable human cell lines (Schmidt et al. 2011). These differences likely stem partially from variations in the interface between FFA2R and the Gα compared to when these are expressed in human cells, and partially from the alternative signaling pathway. Interestingly, based on the maximum activation of the sensor strain being reached at higher concentrations in relation to the LOD compared to human cell lines, the operational range of the developed strain appears increased. Based on this, it is possible that the lower pEC50 found in the developed yeast strain, to some part, can be attributed to it having a wider operational range, as the pEC50 is defined in relation to the maximum activation without taking the slope of the activation curve into account. Moreover, it is relevant to note that results found in human cell lines engineered to have a stable expression of FFA2R are different from those found in primary cells. An example of this can be found in FFA2R activity studies of human neutrophils, where addition of acetate or propionate, respectively, only results in weak activation at concentrations up to 5 mM (Mårtensson et al. 2018, Lind et al. 2020). Even so, these SCFAs become potent activators in the presence of allosteric modulators AZ1729 or Cmp58, stimulating a response at concentrations of 1 mM acetate or 0.025 mM propionate in these cells (Mårtensson et al. 2018, Lind et al. 2020). As a last validation of the performance of our yeast platform, we also compared the pEC50 of propionate activation in our sensor to that of a previously developed growth-based FFA2R sensor in yeast (Brown et al. 2015), and found a slightly lower pEC50 in our platform (ΔpEC50_Pr_ = 0.22).

Other than propionate and acetate, none of the compounds explored in this study had been previously evaluated in a yeast FFA2R platform. The synthetic orthosteric agonist Cmp1 is known to be a more potent agonist of FFA2R compared to the SCFAs in human cell lines, with a 3-fold higher pEC50, and like the SCFAs does not have a bias towards the Gα_i/o_ or Gα_q/11_ subunit families (Hudson et al. 2013, Björkman et al. 2016). In accordance with this, Cmp1 was demonstrated to be a more potent agonist also in our yeast platform, albeit with a slightly lower relative potency compared to acetate and propionate than in human cell lines (Hudson et al. 2013). Comparing the apparent LOD for Cmp1 in stable human cell lines with the LOD for Cmp1 in our platform, we find that these are largely similar for several of the activation assays applied in human cells (Hudson et al. 2013). With this, Cmp1 was verified as a strong activator of Gα_i_-mediated FFA2R signaling and confirmed that the yeast platform could be applied to determine the relative activation strengths of agonists of FFA2R.

Investigating the relative potency of the antagonists/inverse agonists GLPG0974 and CATPB, the yeast sensor, we found that both compounds mediated significant suppression of GFP fluorescence intensity at a concentration of 0.1 µM in the absence and presence of increasing concentrations of propionate (Fig. 5A and B). The one exception was 0.1 µM CATPB at 10 mM propionate, for which suppression was likely undetectable due to sensor saturation by propionate at this concentration. Only a higher concentration of 1 µM CATPB produced significant suppression of activation at this propionate concentration. These results indicate this compound as a weaker antagonist in the yeast platform compared to GLPG0974, which produced a signal suppression at a lower concentration. These results align with the apparent LOD presented for the compounds in stable human cell lines, where a clear signal is produced at corresponding concentrations of GLPG0974 and CATPB (Sergeev et al. 2017). Both compounds have been reported to bind the orthosteric site of FFA2R, but whether they act as antagonists, simply blocking the agonist from binding, or inverse agonists, altering the receptor structure to repress activation, is unclear in the literature (Namour et al. 2016, Sergeev et al. 2016, Miyasato et al. 2023). In the yeast platform developed in this study, it was demonstrated that both GLPG0974 and CATPB suppress GFP fluorescence in the absence of an agonist, indicating a structural change as a result of binding and thereby inverse agonist activity. These results are supported by a recent study in a stable human cell line with constitutively active FFA2R, where both GLPG0974 and CATPB suppressed the receptor activity in the absence of an agonist (Miyasato et al. 2023). They are further in line with several reports from human neutrophils, where both GLPG0974 and CATPB were shown to inhibit transactivation of FFA2R by other receptors and inhibit the allosteric activity by Cmp58 and AZ1729, respectively, indicating structural change upon binding (Lind et al. 2023). GLPG0974 was also indicated as a positive modulator for co-activation by allosteric modulators AZ1729 and Cmp58 in neutrophils (Lind et al. 2022).

Lastly, the allosteric modulators Cmp58 and AZ1729 were evaluated in the yeast platform. While both compounds did act as allosteric agonists, activating the yeast mating pathway at a concentration of 1 µM through Gα_i1_ in absence and presence of propionate, the induction by AZ1729 was weak and only clear at higher concentrations (Fig. 6A and B). Of the two allosteric agonists, Cmp58 was thereby found to induce a higher intensity of fluorescence. While the intensity of response to AZ1729 induction in absence of an agonist is in line with those found for certain outputs in stable human cell lines, this weak response indicates that some ligands may be difficult to detect at lower concentrations in this yeast platform (Bolognini et al. 2016). To further investigate the activity of the compounds in the yeast platform, the effect of simultaneous induction by both was evaluated. With both compounds producing individual activation in the absence and presence of the other, the recent suggestion that Cmp58 and AZ1729 bind different sites in the receptor was further supported (Fig. 6D; Lind et al. 2020). Conversely, no cooperative effects, i.e. additional increase in signal intensity above that produced by the individual compounds, were found. While this is in contrast with the reported cooperative activation in neutrophils, the mechanisms behind this effect are still unclear and have been hypothesized to occur either via the activated G protein subunits or independently of the major signaling routes downstream of FFA2R (Lind et al. 2020, 2022). As such, the absence of a cooperative activation in the yeast platform further suggests that this effect occurs separately from the Gα_i_-based signaling.

Altogether, the results presented in this work demonstrate the applicability of the developed yeast-based FFA2R sensor for evaluation of ligands acting through Gα_i1_, with potential for applications in ligand screenings. Applying a streamlined mating pathway for sensing with GFP as an output enabled both tuning of the pathway response and a shortened incubation time. This allows for an increased efficiency of screening compared to previously developed yeast sensors for FFA2R, potentially applicable investigation of large compound libraries in search of novel FFA2R ligands acting through the Gα_i_ subunit family. The strain captures the activation by agonists and allosteric agonists, and suppression by inverse agonists, and can contribute with insights into the cooperative activation by the allosteric agonists. Moreover, we demonstrate the first FFA2R yeast sensor with near to no baseline activity. While it was not further evaluated in this study, this strain would be applicable for screening of FFAR2 mutant libraries towards identification of key amino acid positions and binding sites of agonists of interest. With this, we present an improved strain for screening of FFA2R ligands, with potential for broader future applications.

## Supplementary Material

foaf001_Supplemental_File

## Data Availability

Raw data and scripts used in this study are available at https://github.com/AndreaClausenLind/FFA2Ranalysis.
