# Supplementary material for: Development of a yeast-based sensor platform for evaluation of ligands recognized by the human free fatty acid 2 receptor
Source: FEMS Yeast Res. 2025 Jan 17;25:foaf001. doi: 10.1093/femsyr/foaf001 (PMC11781196; doi:10.1093/femsyr/foaf001)
Supplement: foaf001_Supplemental_File [file foaf001_supplemental_file.docx]

**Supplementary information**


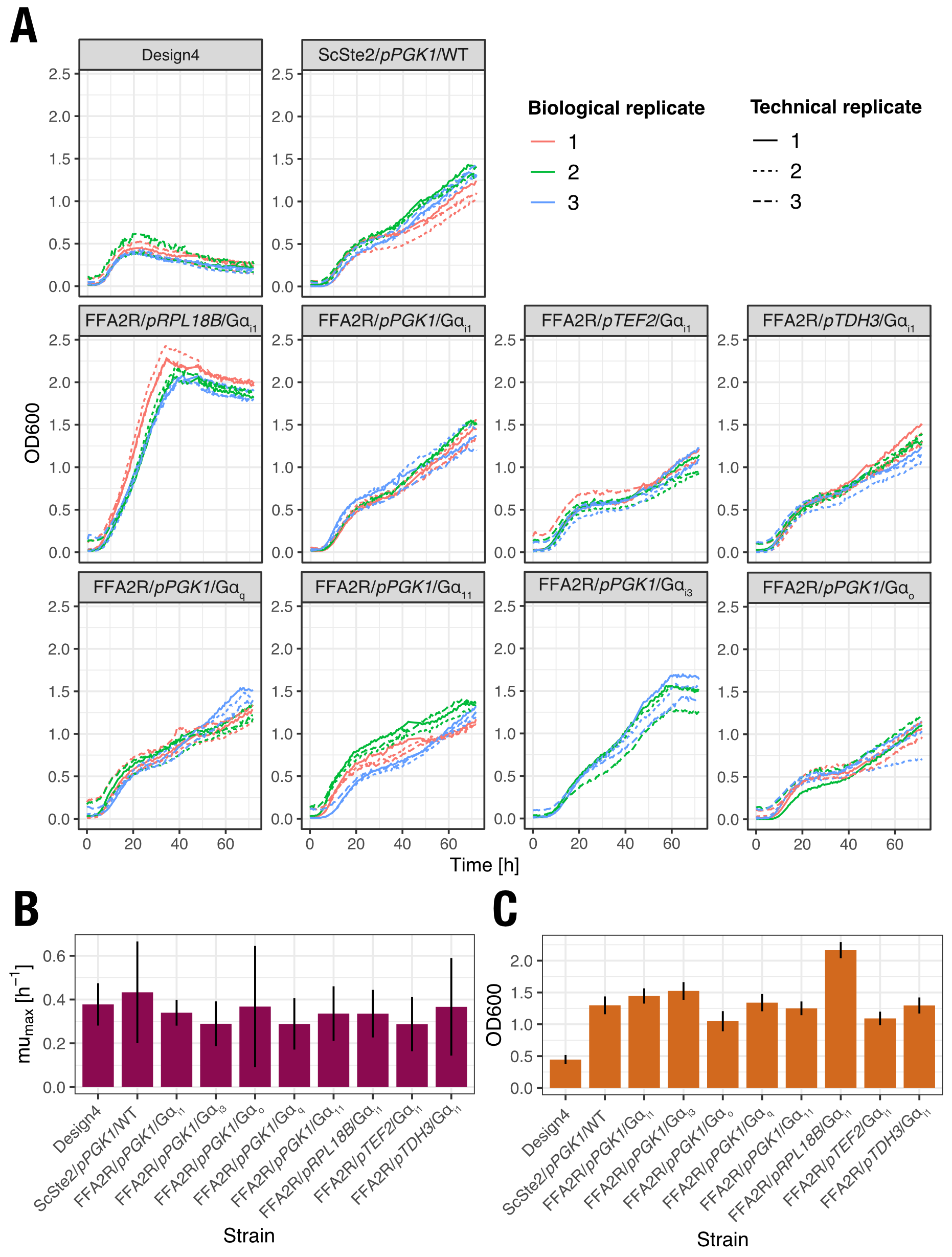


**Figure S1.** **Growth curves and growth parameters of strains in study.** The plots display the growth curves for each biological and technical replicate (A) and parameters of these. The growth rate (B) of strains was calculated from the beginning of the exponential growth phase for each replicate, with bars presenting the average of replicates (n=6) and vertical lines the s.d. of these. The maximal OD_600_ (C) of strains was extracted for each replicate during the 50 h strains were analyzed, with bars presenting the average of replicates (n=6) and vertical lines the s.d. of these.


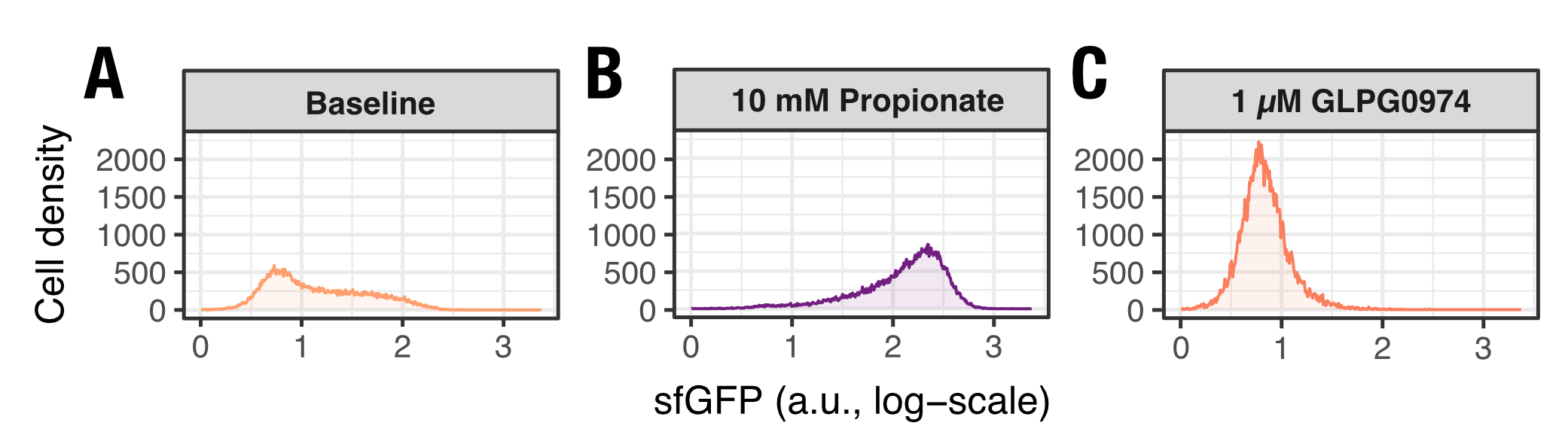


**Figure S2.** **Distribution of raw flow cytometry data**. The plots display cell density defined as the distribution of GFP intensity per cell in a population of 15000 cells after 3 h of induction, combining the data of three biological replicates per condition. Three different conditions were evaluated in the same run, including (A) distribution of an uninduced population (baseline), (B) distribution of a population induced with the natural agonist propionate at 10 mM, and (C) inhibition by the antagonist GLPG0974 at 1 μM.

**Table S1.** Plasmids used in this study.

| *Golden gate assembled plasmids* | | | |  |
| --- | --- | --- | --- | --- |
| Plasmid name | Level | Assembled from | Expression cassettes specifics | Origin |
| pMC-3-GPA1 | 0 | pYTK001, GPA1_p3 |  | This study |
| pMC-3-ScSTE2 | 0 | pYTK001, ScSTE2_p3 |  | This study |
| pMC-3-GPA1(i1) | 0 | pYTK001, *GPA1*(1-467), G(alpha)(i1) C-term |  | This study |
| pMC-3-GPA1(i3) | 0 | pYTK001, *GPA1*(1-467), G(alpha)(i3) C-term |  | This study |
| pMC-3-GPA1(o) | 0 | pYTK001, *GPA1*(1-467), G(alpha)(o) C-term |  | This study |
| pMC-3-GPA1(q) | 0 | pYTK001, *GPA1*(1-467), G(alpha)(q) C-term |  | This study |
| pMC-3-GPA1(11) | 0 | pYTK001, *GPA1*(1-467), G(alpha)(11) C-term |  | This study |
| pMC-3-HIS3 | 0 | pYTK001, HIS3_p3 |  | This study |
| pMC-2-LexOx6LEU2p | 0 | pYTK001, LexOx6_LEU2p_p2 |  | This study |
| pMC-3-miRFP670 | 0 | pYTK001, miRFP670_p3 |  | (Scott *et al.* 2022) |
| pMC-3-FFA2R | 0 | pYTK001, FFA2R_p3 |  | This study |
| LS-HIS3-R1 | 1 | pYTK095, pYTK002, pMC-2-LexOx6LEU2p, pMC-3-miRFP670, pYTK051, pYTK067 |  | This study |
| L1-miRFP670-RE | 1 | pYTK095, pYTK003, pYTK014, pMC-3-miRFP670, pYTK053, pYTK072 | *L1-pTEF2-miRFP670-tADH1-RE* | This study |
| pMC-XI2_HIS3-miRFP670 | 2 | pMC-XI2, LS-HIS3-R1, L1-miRFP670-RE | *pMC-XI2_LexO6xLEU2p-HIS3-tENO1-TEF2P-miRFP670-tADH1* | This study |
| pMC-XII2-ScSTE2 | 2 | pMC-XII2,pYTK010, pMC-3-ScSTE2, pYTK052 | *pMC-XII2_pCCW12-ScSTE2-tSSA1* |  |
| pMC-XI5-GPA1 | 2 | pMC-XI5, pYTK011, pMC-3-GPA1, pYTK055 | *pMC-XI5_pPGK1-GPA1-tENO2* |  |
| pMC-XI5_GPA1(i1) | 2 | pMC-XI5, pYTK011, pMC-3-GPA1(i1), pYTK055 | *pMC-XI5_pPGK1-GPA1(i1)-tENO2* | This study |
| pMC-XI5_GPA1(i3) | 2 | pMC-XI5, pYTK011, pMC-3-GPA1(i3), pYTK055 | *pMC-XI5_pPGK1-GPA1(i3)-tENO2* | This study |
| pMC-XI5_GPA1(o) | 2 | pMC-XI5, pYTK011, pMC-3-GPA1(o), pYTK055 | *pMC-XI5_pPGK1-GPA1(o)-tENO2* | This study |
| pMC-XI5-GPA1(i1)_h1 | 2 | pMC-XI5, pYTK017, pMC-3-GPA1(i1), pYTK055 | *pMC-XI5_pRPL18B-GPA1(i1)-tENO2* |  |
| pMC-XI5-GPA1(i1)_h2 | 2 | pMC-XI5, pYTK014, pMC-3-GPA1(i1), pYTK055 | *pMC-XI5_pTEF2-GPA1(i1)-tENO2* |  |
| pMC-XI5-GPA1(i1)_h3 | 2 | pMC-XI5, pYTK009, pMC-3-GPA1(i1), pYTK055 | *pMC-XI5_pTDH3-GPA1(i1)-tENO2* |  |
| pMC-XI5-GPA1(q) | 2 | pMC-XI5, pYTK011, pMC-3-GPA1(q), pYTK055 | *pMC-XI5_pPGK1-GPA1(q)-tENO2* |  |
| pMC-XI5-GPA1(11) | 2 | pMC-XI5, pYTK011, pMC-3-GPA1(11), pYTK055 | *pMC-XI5_pPGK1-GPA1(11)-tENO2* |  |
| XII-1_miRFP670 | 2 |  | *pMC-XII1_pTEF1-miRFP670-tCYC1* | (Scott *et al.* 2022) |
| pMC-XII2-FFA2R | 2 | pMC-XII2, pYTK010, pMC-3-FFA2R, pYTK052 | *pMC-XII2_pCCW12-FFA2R-tSSA1* | This study |
| *Cas9/gRNA plasmids* | | | |  |
| *Plasmid name* | *Genomic target* | *gRNA* | - | Origin |
| pCas9_KanMX_gRNA |  |  |  | (Hedin *et al.* 2023) |
| Cas9KanMX_XI2 | XI-2 | GTTGACCAGTTGATCAGTTG |  | This study |
| Cas9KanMX_XI5 | XI-5 | TGAGAATACTGTTGTAAAAC |  | This study |
| Cas9KanMX_XII2 | XII-2 | TGAAACTCTAATCCTACTAT |  | This study |
| Cas9KanMX_XI1.S288C | XI1 (S288C) | GCGGTGCACGGATTTCAGCA |  | This study |
| Cas9KanMX_GPA1 | GPA1 | GTTTTGCTGGATGATTAGAT |  | This study |
| Cas9KanMX_STE2 | STE2 | TTGCAACTCATCGAAAGTGA |  | This study |

**Table S2.** Oligonucleotides used in this study.

| *Oligo name* | *Sequence* | *Description* |
| --- | --- | --- |
| *Oligos used in cloning of genes into Lv 0 MoClo plasmids* | | |
| fw_STE2_p3 | gcatcgtctcatcggtctcaTATGTCTGATGCGGCTCC | Template: Design 4 genome.  Product: ScSTE2_p3  Amplifying *ScSTE2* with part 3 overhangs. |
| rv_STE2_p3 | atgccgtctcaggtctcaGGATCCTAAATTATTATTATCTTCAGTC |  |
| fw_HIS3_p3 | gcatcgtctcatcggtctcatATGACAGAGCAGAAAGCCCTAG | Template: IMX581 genome.  Product: HIS3_p3  Amplify *HIS3* with part 3 overhangs. |
| rv_HIS3_p3 | atgccgtctcaggtctcaggatccCTACATAAGAACACCTTTGGTG |  |
| fw_LexOLEUp_p2 | gcatcgtctcatcggtctcaAACGGGTAGTCCATCGTTG | Template: Design 4 genome.  Product: LexOx6_LEU2p_p2  Amplifying *pLexO6xLEU2* promoter with part 2 overhangs. |
| rv_LexOLEUp_p2 | atgccgtctcaggtctcaCATAGATCTTAGAATGGTATATCCTTG |  |
| fw_GPA1_p3 | gcatcgtctcatcggtctcaTATGGGGTGTACAGTGAGTAC | Template: Design 4 genome.  Products: GPA1(1-467) or GPA1_p3 depending on the reverse primer.  Amplifying *GPA1* with or without the 5 C-terminal amino acids, with overhang for chimeric C-terminal attachment. Part 3 or 3c overhangs. |
| rv_GPA1-MoClo-Chimera | ATGCcgtctcaTTTCAGGTTTTGCTGGATGATTAG |  |
| rv_GPA1_p3 | atgccgtctcaggtctcaggatccGTTAAATAATACCAATTTTTTTCAGG |  |
| fw_FFA2R_p3 | gcatcgtctcatcggtctcaTATGCTTCCTGATTGGAAGAGTAG | Template: *FFA2R* gBlock.  Product: FFA2R_p3  Amplifying *FFA2R* with part 3 overhangs. |
| rv_FFA2R_p3 | atgccgtctcaggtctcaggatccGTTACTCGGTAGTGAAGTCGCTG |  |
| *Oligos annealed and used directly in MoClo assembly* | | |
| C-term_G(alpha)i1-U | GAAAGATTGTGGTTTGTTTTGAGGATCCTGA | Product: G(alpha)(i1) C-term  Oligos for C-terminal replacement of *GPA1* using MoClo assembly, part 3c and 3 overhangs |
| C-term_G(alpha)i1-D | GGTCTCAGGATCCTCAAAACAAACCACAATC |  |
| C-term_G(alpha)i3-U | GAAAGAATGTGGTTTGTACTGAGGATCCTGA | Product: G(alpha)(i3) C-term  Oligos for C-terminal replacement of *GPA1* using GoldenGate, part 3c and 3 overhangs |
| C-term_G(alpha)i3-D | GGTCTCAGGATCCTCAGTACAAACCACATTC |  |
| C-term_G(alpha)o-U | GAAAGGTTGTGGCTTGTACTGAGGATCCTGA | Product: G(alpha)(o) C-term  Oligos for C-terminal replacement of *GPA1* MoClo assembly, part 3c and 3 overhangs |
| C-term_G(alpha)o-D | GGTCTCAGGATCCTCAGTACAAGCCACAACC |  |
| C-term_G(alpha)11-U | GAAAGAATACAACCTATATTGAGGATCCTGA | Product: G(alpha)(11) C-term  Oligos for C-terminal replacement of *GPA1* MoClo assembly, part 3c and 3 overhangs |
| C-term_G(alpha)11-D | GGTCTCAGGATCCTCAATATAGGTTGTATTC |  |
| C-term_G(alpha)q-U | GAAAGAATACAACCTAGTTTGAGGATCCTGA | Product: G(alpha)(q) C-term  Oligos for C-terminal replacement of *GPA1* MoClo assembly, part 3c and 3 overhangs |
| C-term_G(alpha)q-D | GGTCTCAGGATCCTCAAACTAGGTTGTATTC |  |
| *Oligos used in colony PCR* | | |
| fw_cPCR_XI-2_EC | GTTTGTAGTTGGCGGTGGAG | Template: Design 4 genome. Colony PCR of integration site *XI-2* |
| rv_cPCR_XI-2_EC | GAGACAAGATGGGGCAAGAC |  |
| rv_cPCR_XI-5_EC | GCATGGTCACCGCTATCAGC | Template: Design 4 genome. Colony PCR of integration site *XI-5* |
| fw_cPCR_XI-5_EC | CTCAATGATCAAAATCCTGAATGCA |  |
| rv_cPCR_XII-1_EC | GGACGACAACTACGGAGGAT | Template: Design 4 genome. Colony PCR of integration site *XII-1* |
| fw_cPCR_XII-1_EC | CTGGCAAGAGAACCACCAAT |  |
| rv_cPCR_XII-2_EC | GGCCCTGATAAGGTTGTTG | Template: Design 4 genome. Colony PCR of integration site *XII-2* |
| fw_cPCR_XII-2_EC | CGAAGAAGGCCTGCAATTC |  |
| rv_cPCR_GPA1/STE2(D4) | gagaggattatcaactgctaagcc | Template: Design 4 genome. Colony PCR of *STE2* and *GPA1* in strain Design 4. Pair with fw_colPCR_STE2(D4). |
| fw_cPCR_STE2(D4) | ggttcatcatctcatggatctgc | Template: Design 4 genome. Colony PCR of *STE2* in strain Design 4 |
| rv_cPCR_STE2(D4) | gcaaacaaatcacgagcgac |  |
| fw_colPCR_URA3(D4) | GCAACCGGATCCtaactc | Template: Design 4 genome. Colony PCR of integration site *URA3* |
| rv_colPCR_URA3(D4) | gttacttggttctggcgag |  |
| *Oligos for knockout of genes* | | |
| URA3(D4)KO-U | GACGCGTGTATCCGCCCGCTCTTTTGGTCACCCATGTATGCTGAGCAGTTACAGAGATGTAGAGCACTTGAATCCACTGCCCCGGGAATCTCGGTCGTAATGATTTCTATAATGACGAAA | Knockout of *URA3* in strain Design 4 |
| URA3(D4)KO-D | TTTCGTCATTATAGAAATCATTACGACCGAGATTCCCGGGGCAGTGGATTCAAGTGCTCTACATCTCTGTAACTGCTCAGCATACATGGGTGACCAAAAGAGCGGGCGGATACACGCGTC |  |
| STE2(D4)KO-U | CTATAATTATAATTGGTTACTTAAAAATGCACCGTTAAGAACCATATCCAAGAATCAAAATGATCAAAATTTACGGCTTTGAAAAAGTAATTTCGTGACCTTCGGTATAAGGTTACTACT | Knockout of *STE2* in strain Design 4 |
| STE2(D4)KO-D | AGTAGTAACCTTATACCGAAGGTCACGAAATTACTTTTTCAAAGCCGTAAATTTTGATCATTTTGATTCTTGGATATGGTTCTTAACGGTGCATTTTTAAGTAACCAATTATAATTATAG |  |
| GPA1(D4)KO-U | CAAAGAAGGTTAATGTGGCTGTGGTTTCAGGGTCCATAAAGCCCACATGGATAACATTACGCCTTGTGAAATTGCAAATATGGTGATTTGAAACGTTTCCTAGTGCAGCAGGATCACAGA | Knockout of *GPA1* in strain Design 4 |
| GPA1(D4)KO-D | TCTGTGATCCTGCTGCACTAGGAAACGTTTCAAATCACCATATTTGCAATTTCACAAGGCGTAATGTTATCCATGTGGGCTTTATGGACCCTGAAACCACAGCCACATTAACCTTCTTTG |  |
| *Oligos used for cloning of gRNAs into Cas9KanMX* | | |
| fw_Cas9KanMX_gRNA-U | ggaatttaatcgcggcctc | Template: pCas9KanMX_gRNA plasmid. |
| rv_gRNAplas_John | gatcatttatctttcactgcggagaag |  |
| rv_Cas9KanmX_gRNA-D | ggtccttttcatcacgtgc | Template: pCas9KanMX_gRNA plasmid. Pair with fw_gRNAplas_gRNA primers. |
| fw_gRNAplas_GPA1_1 | ctccgcagtgaaagataaatgatcACAGTGAGTACGCAAACAATgttttagagctagaaatagcaag |  |
| fw_gRNAplas_GPA1_2 | ctccgcagtgaaagataaatgatcGTTTTGCTGGATGATTAGATgttttagagctagaaatagcaag |  |
| fw_gRNAplas_STE2_1 | ctccgcagtgaaagataaatgatcTTGCAACTCATCGAAAGTGAgttttagagctagaaatagcaag |  |
| fw_gRNAplas_STE2_2 | ctccgcagtgaaagataaatgatcGCTGATGCAAGTTACAAAGAgttttagagctagaaatagcaag |  |
| fw_rRNAplas_URA3_1 | ctccgcagtgaaagataaatgatcTCCTTGAAATTTTTTTGATTgttttagagctagaaatagcaag |  |
| fw_rRNAplas_URA3_2 | ctccgcagtgaaagataaatgatcGAGGACTATTTGCAAAGGGAgttttagagctagaaatagcaag |  |
| fw_gRNAplas_XI-1.S288C | ctccgcagtgaaagataaatgatcGCGGTGCACGGATTTCAGCAgttttagagctagaaatagcaag |  |
| fw_gRNAplas_XI-2 | ctccgcagtgaaagataaatgatcGTTGACCAGTTGATCAGTTGgttttagagctagaaatagcaag |  |
| fw_gRNAplas_gXI-5 | cttctccgcagtgaaagataaatgatcTGAGAATACTGTTGTAAAACgttttagagctagaaatagcaag |  |
| fw_gRNAplas_XII-2 | ctccgcagtgaaagataaatgatcTGAAACTCTAATCCTACTATgttttagagctagaaatagcaag |  |

**Table S3. Part sequences, inserted in the pYTK001** (Lee *et al.* 2015) **plasmid backbone.** Grey hightlight - BsaI recognition site. Green hightlight - start codon. Red highlight - stop codon. Bold - BsaI generated overhang. Orange - promoter sequence. Purple - terminator sequence. Blue - mutated sequence. Italic - chimeric C-terminus of Gpa1.

| **Name** | **Sequence** | **Description** |
| --- | --- | --- |
| FFA2R_p3 | ggtctca**tATG**CTTCCTGATTGGAAGAGTAGCTTAATCCTAATGGCGTATATAATTATCTTCCTAACGGGACTTCCAGCTAACTTGTTAGCGCTGAGGGCATTCGTCGGTAGAATACGTCAGCCTCAACCGGCACCCGTTCATATTCTATTGTTGAGCCTAACGTTAGCTGACTTGCTATTATTACTATTACTTCCGTTCAAGATTATTGAGGCAGCTTCTAATTTCAGATGGTACTTGCCAAAAGTAGTCTGTGCGTTGACGTCCTTTGGTTTTTACAGTAGTATATACTGCTCTACCTGGCTACTGGCGGGGATCAGCATCGAAAGGTATTTAGGTGTTGCCTTTCCCGTTCAATACAAGTTGTCTCGTAGACCTTTATATGGAGTAATAGCAGCGTTAGTCGCCTGGGTGATGTCATTCGGTCATTGCACAATCGTTATCATTGTCCAGTACCTTAATACCACCGAACAAGTAAGGTCCGGCAATGAGATCACCTGTTACGAGAATTTTACCGATAACCAACTTGACGTTGTCTTGCCAGTAAGACTGGAGCTGTGCCTAGTACTGTTTTTCATTCCTATGGCAGTGACCATTTTCTGCTATTGGAGATTTGTCTGGATCATGCTGAGCCAACCGCTAGTTGGTGCACAACGTCGTAGACGTGCTGTGGGGTTAGCAGTTGTCACCCTGTTGAACTTCCTAGTGTGTTTTGGCCCCTATAACGTCAGTCACTTGGTTGGCTATCACCAGAGGAAGTCACCTTGGTGGAGATCAATCGCGGTCGTATTTTCTAGTTTGAACGCGTCACTTGATCCCCTACTTTTTTATTTTAGCAGCTCAGTAGTGAGGCGTGCATTCGGGAGGGGGCTTCAAGTGCTTCGTAACCAAGGGAGTTCACTGTTAGGCCGTCGTGGCAAGGATACCGCGGAAGGGACTAATGAGGATCGTGGGGTAGGCCAAGGAGAAGGTATGCCTTCCAGCGACTTCACTACCGAGTAAcgg**atcc**tgagacc | Human *FFA2R* (UniProt ID: O15552) cloned into pYTK001 with BasI overhangs compatible with part 2 and 4 of the MoClo cloning toolkit. |
| ScSTE2_p3 | ggtctca**tATG**TCTGATGCGGCTCCTTCATTGAGCAATCTATTTTATGATCCAACGTATAATCCTGGTCAAAGCACCATTAACTACACTTCCATATATGGGAATGGATCTACCATCACTTTCGATGAGTTGCAAGGTTTAGTTAACAGTACTGTTACTCAGGCCATTATGTTTGGTGTCAGATGTGGTGCAGCTGCTTTGACTTTGATTGTCATGTGGATGACATCGAGAAGCAGAAAAACGCCGATTTTCATTATCAACCAAGTTTCATTGTTTTTAATCATTTTGCATTCTGCACTCTATTTTAAATATTTACTGTCTAATTACTCTTCAGTGACTTACGCTCTCACCGGATTTCCTCAGTTCATCAGTAGAGGTGACGTTCATGTTTATGGTGCTACAAATATAATTCAAGTCCTTCTTGTGGCTTCTATTGAGACTTCACTGGTGTTTCAGATAAAAGTTATTTTCACAGGCGACAACTTCAAAAGGATAGGTTTGATGCTGACGTCGATATCTTTCACTTTAGGGATTGCTACAGTTACCATGTATTTTGTAAGCGCTGTTAAAGGTATGATTGTGACTTATAATGATGTTAGTGCCACCCAAGATAAATACTTCAATGCATCCACAATTTTACTTGCATCCTCAATAAACTTTATGTCATTTGTCCTGGTAGTTAAATTGATTTTAGCTATTAGATCAAGAAGATTCCTTggtctTAAGCAGTTCGATAGTTTCCATATTTTACTCATAATGTCATGTCAATCTTTGTTGGTTCCATCGATAATATTCATCCTCGCATACAGTTTGAAACCAAACCAGGGAACAGATGTCTTGACTACTGTTGCAACATTACTTGCTGTATTGTCTTTACCATTATCATCAATGTGGGCCACGGCTGCTAATAATGCATCCAAAACAAACACAATTACTTCAGACTTTACAACATCCACAGATAGGTTTTATCCAGGCACGCTGTCTAGCTTTCAAACTGATAGTATCAACAACGATGCTAAAAGCAGTCTCAGAAGTAGATTATATGACCTATATCCTAGAAGGAAGGAAACAACATCGGATAAACATTCGGAAAGAACTTTTGTTTCTGAGACTGCAGATGATATAGAGAAAAATCAGTTTTATCAGTTGCCCACACCTACGAGTTCAAAAAATACTAGGATAGGACCGTTTGCTGATGCAAGTTACAAAGAGGGAGAAGTTGAACCCGTCGACATGTACACTCCCGATACGGCAGCTGATGAGGAAGCCAGAAAGTTCTGGACTGAAGATAATAATAATTTATGA**atcc**tgagacc | *ScSTE2* sequence amplified from genome of strain Design 4 cloned into pYTK001 with BasI overhangs compatible with part 2 and 4 of the MoClo cloning toolkit (Lee *et al.* 2015). |
| LexO6xLEU2p_p2 | ggtctca**AACG**GGTAGTCCATCGTTGTAGGATACTGTATATACACCCAGTAGAGTAGGTGACTACTGTATGAGCATACAGTAGTGACAACCACTTACTGTATATAAATACAGTAGTGGTCATCGATACTGTATATAAAACCAGTAGGCAATCGTTTGTACTGTATGTACATACAGTATACCTCGCAACTACTGTATATAAACACAGTAATTAACTTGTAATATTCTAATCAATTGACAATATTATTTAAGGACCTATTGTTTTTTCCAATAGGTGGTTAGCAATCGTCTTACTTTCTAACTTTTCTTACCTTTTACATTTCAGCAATATATATATATATATTTCAAGGATATACCATTCTAAGATC**TATG**tgagacc | *LexO6xLEU2p* promoter sequence amplified from genome of strain Design 4 cloned into pYTK001 with BasI overhangs compatible with part 1 and 3 of the MoClo cloning toolkit (Lee *et al.* 2015). |
| GPA1_p3 | ggtctca**tATG**GGGTGTACAGTGAGTACGCAAACAATAGGTGACGAAAGTGATCCTTTTCTACAGAACAAAAGAGCCAATGATGTCATCGAGCAATCGTTGCAGCTGGAGAAACAACGTGACAAGAATGAAATAAAACTGTTACTATTAGGTGCCGGTGAGTCAGGTAAATCAACGGTTTTAAAACAATTAAAATTATTACATCAAGGCGGTTTCTCCCATCAAGAAAGGTTACAGTATGCTCAAGTGATATGGGCAGATGCCATACAATCAATGAAAATTTTGATTATTCAGGCCAGAAAACTAGGTATTCAACTTGACTGTGATGATCCGATCAACAATAAAGATTTGTTTGCATGCAAGAGAATACTGCTAAAGGCTAAAGCTTTAGATTATATCAACGCCAGTGTTGCCGGTGGTTCTGATTTTCTAAATGATTATGTACTGAAGTACTCAGAAAGGTATGAAACTAGGAGGCGTGTTCAGAGTACCGGACGAGCAAAAGCTGCTTTCGATGAGGACGGAAATATTTCTAATGTCAAAAGTGACACTGACAGAGATGCTGAAACGGTGACGCAAAATGAGGATGCTGATAGAAACAACAGTAGTAGAATTAACCTACAGGATATTTGCAAGGACTTGAACCAAGAAGGCGATGACCAGATGTTTGTTAGAAAAACATCAAGGGAAATTCAAGGACAAAATAGACGAAATCTTATTCACGAGGACATTGCTAAGGCAATAAAGCAACTTTGGAATAACGACAAAGGTATAAAGCAGTGTTTTGCACGTTCTAATGAGTTTCAATTGGAGGGCTCAGCTGCATACTACTTTGATAACATTGAGAAATTTGCTAGTCCGAATTATGTCTGTACGGATGAGGACATTTTGAAGGGCCGTATAAAGACTACAGGCATTACAGAAACCGAATTTAACATCGGCTCGTCCAAATTCAAGGTTCTCGACGCTGGTGGGCAGCGTTCTGAACGTAAGAAGTGGATTCATTGTTTCGAAGGAATTACAGCAGTTTTATTTGTTTTAGCAATGAGTGAATACGACCAGATGTTGTTTGAGGATGAAAGAGTGAACAGAATGCATGAATCAATAATGCTATTTGACACGTTATTGAACTCTAAGTGGTTCAAAGATACACCGTTTATTTTGTTTTTAAATAAAATTGATTTGTTCGAGGAAAAGGTAAAAAGCATGCCCATAAGAAAGTACTTTCCTGATTACCAGGGACGTGTCGGCGATGCAGAAGCGGGTCTAAAATATTTTGAGAAGATATTTTTGAGCTTGAATAAGACAAACAAACCAATCTACGTGAAACGAACCTGCGCTACCGATACCCAAACTATGAAGTTCGTATTGAGTGCAGTCACCGATCTAATCATCCAGCAAAACCTGAAAAAAATTGGTATTATTTAAcgg**atcc**tgagacc | *GPA1* (*S. cerevisiae)* was amplified from genome of strain Design 4 and cloned into pYTK001 by BsaI digestion together with annealed oligos containing BsaI compatible overhangs. The resulting plasmid contains the gene with part 3 adaptors the MoClo cloning toolkit (Lee *et al.* 2015). |
| GPA1(i1)_p3 | ggtctca**tATG**GGGTGTACAGTGAGTACGCAAACAATAGGTGACGAAAGTGATCCTTTTCTACAGAACAAAAGAGCCAATGATGTCATCGAGCAATCGTTGCAGCTGGAGAAACAACGTGACAAGAATGAAATAAAACTGTTACTATTAGGTGCCGGTGAGTCAGGTAAATCAACGGTTTTAAAACAATTAAAATTATTACATCAAGGCGGTTTCTCCCATCAAGAAAGGTTACAGTATGCTCAAGTGATATGGGCAGATGCCATACAATCAATGAAAATTTTGATTATTCAGGCCAGAAAACTAGGTATTCAACTTGACTGTGATGATCCGATCAACAATAAAGATTTGTTTGCATGCAAGAGAATACTGCTAAAGGCTAAAGCTTTAGATTATATCAACGCCAGTGTTGCCGGTGGTTCTGATTTTCTAAATGATTATGTACTGAAGTACTCAGAAAGGTATGAAACTAGGAGGCGTGTTCAGAGTACCGGACGAGCAAAAGCTGCTTTCGATGAGGACGGAAATATTTCTAATGTCAAAAGTGACACTGACAGAGATGCTGAAACGGTGACGCAAAATGAGGATGCTGATAGAAACAACAGTAGTAGAATTAACCTACAGGATATTTGCAAGGACTTGAACCAAGAAGGCGATGACCAGATGTTTGTTAGAAAAACATCAAGGGAAATTCAAGGACAAAATAGACGAAATCTTATTCACGAGGACATTGCTAAGGCAATAAAGCAACTTTGGAATAACGACAAAGGTATAAAGCAGTGTTTTGCACGTTCTAATGAGTTTCAATTGGAGGGCTCAGCTGCATACTACTTTGATAACATTGAGAAATTTGCTAGTCCGAATTATGTCTGTACGGATGAGGACATTTTGAAGGGCCGTATAAAGACTACAGGCATTACAGAAACCGAATTTAACATCGGCTCGTCCAAATTCAAGGTTCTCGACGCTGGTGGGCAGCGTTCTGAACGTAAGAAGTGGATTCATTGTTTCGAAGGAATTACAGCAGTTTTATTTGTTTTAGCAATGAGTGAATACGACCAGATGTTGTTTGAGGATGAAAGAGTGAACAGAATGCATGAATCAATAATGCTATTTGACACGTTATTGAACTCTAAGTGGTTCAAAGATACACCGTTTATTTTGTTTTTAAATAAAATTGATTTGTTCGAGGAAAAGGTAAAAAGCATGCCCATAAGAAAGTACTTTCCTGATTACCAGGGACGTGTCGGCGATGCAGAAGCGGGTCTAAAATATTTTGAGAAGATATTTTTGAGCTTGAATAAGACAAACAAACCAATCTACGTGAAACGAACCTGCGCTACCGATACCCAAACTATGAAGTTCGTATTGAGTGCAGTCACCGATCTAATCATCCAGCAAAACCTGAAA*GATTGTGGTTTGTTTTGA*gg**atcc**tgagacc | *GPA1-G(alpha)i1* chimeric protein constructed by truncating the 5 C-terminal amino acids of *GPA1* (*S. cerevisiae)* and replacing these with those of human *G(alpha)i1* (Brown *et al.* 2003). *GPA1* was amplified from genome of strain Design 4 and cloned into pYTK001 by BsaI digestion together with annealed oligos containing BsaI compatible overhangs. The resulting plasmid contains the gene with part 3 adaptors the MoClo cloning toolkit (Lee *et al.* 2015). |
| GPA1(i3)_p3 | ggtctca**TATG**GGGTGTACAGTGAGTACGCAAACAATAGGTGACGAAAGTGATCCTTTTCTACAGAACAAAAGAGCCAATGATGTCATCGAGCAATCGTTGCAGCTGGAGAAACAACGTGACAAGAATGAAATAAAACTGTTACTATTAGGTGCCGGTGAGTCAGGTAAATCAACGGTTTTAAAACAATTAAAATTATTACATCAAGGCGGTTTCTCCCATCAAGAAAGGTTACAGTATGCTCAAGTGATATGGGCAGATGCCATACAATCAATGAAAATTTTGATTATTCAGGCCAGAAAACTAGGTATTCAACTTGACTGTGATGATCCGATCAACAATAAAGATTTGTTTGCATGCAAGAGAATACTGCTAAAGGCTAAAGCTTTAGATTATATCAACGCCAGTGTTGCCGGTGGTTCTGATTTTCTAAATGATTATGTACTGAAGTACTCAGAAAGGTATGAAACTAGGAGGCGTGTTCAGAGTACCGGACGAGCAAAAGCTGCTTTCGATGAGGACGGAAATATTTCTAATGTCAAAAGTGACACTGACAGAGATGCTGAAACGGTGACGCAAAATGAGGATGCTGATAGAAACAACAGTAGTAGAATTAACCTACAGGATATTTGCAAGGACTTGAACCAAGAAGGCGATGACCAGATGTTTGTTAGAAAAACATCAAGGGAAATTCAAGGACAAAATAGACGAAATCTTATTCACGAGGACATTGCTAAGGCAATAAAGCAACTTTGGAATAACGACAAAGGTATAAAGCAGTGTTTTGCACGTTCTAATGAGTTTCAATTGGAGGGCTCAGCTGCATACTACTTTGATAACATTGAGAAATTTGCTAGTCCGAATTATGTCTGTACGGATGAGGACATTTTGAAGGGCCGTATAAAGACTACAGGCATTACAGAAACCGAATTTAACATCGGCTCGTCCAAATTCAAGGTTCTCGACGCTGGTGGGCAGCGTTCTGAACGTAAGAAGTGGATTCATTGTTTCGAAGGAATTACAGCAGTTTTATTTGTTTTAGCAATGAGTGAATACGACCAGATGTTGTTTGAGGATGAAAGAGTGAACAGAATGCATGAATCAATAATGCTATTTGACACGTTATTGAACTCTAAGTGGTTCAAAGATACACCGTTTATTTTGTTTTTAAATAAAATTGATTTGTTCGAGGAAAAGGTAAAAAGCATGCCCATAAGAAAGTACTTTCCTGATTACCAGGGACGTGTCGGCGATGCAGAAGCGGGTCTAAAATATTTTGAGAAGATATTTTTGAGCTTGAATAAGACAAACAAACCAATCTACGTGAAACGAACCTGCGCTACCGATACCCAAACTATGAAGTTCGTATTGAGTGCAGTCACCGATCTAATCATCCAGCAAAACCTGAAA*GAATGTGGTTTGTACTGA*gg**atcc**tgagacc | *GPA1-G(alpha)i3* chimeric protein constructed by truncating the 5 C-terminal amino acids of *GPA1* (*S. cerevisiae)* and replacing these with those of human *G(alpha)i3* (Brown *et al.* 2003). *GPA1* was amplified from genome of strain Design 4 and cloned into pYTK001 by BsaI digestion together with annealed oligos containing BsaI compatible overhangs. The resulting plasmid contains the gene with part 3 adaptors the MoClo cloning toolkit (Lee *et al.* 2015). |
| GPA1(o)_p3 | ggtctca**TATG**GGGTGTACAGTGAGTACGCAAACAATAGGTGACGAAAGTGATCCTTTTCTACAGAACAAAAGAGCCAATGATGTCATCGAGCAATCGTTGCAGCTGGAGAAACAACGTGACAAGAATGAAATAAAACTGTTACTATTAGGTGCCGGTGAGTCAGGTAAATCAACGGTTTTAAAACAATTAAAATTATTACATCAAGGCGGTTTCTCCCATCAAGAAAGGTTACAGTATGCTCAAGTGATATGGGCAGATGCCATACAATCAATGAAAATTTTGATTATTCAGGCCAGAAAACTAGGTATTCAACTTGACTGTGATGATCCGATCAACAATAAAGATTTGTTTGCATGCAAGAGAATACTGCTAAAGGCTAAAGCTTTAGATTATATCAACGCCAGTGTTGCCGGTGGTTCTGATTTTCTAAATGATTATGTACTGAAGTACTCAGAAAGGTATGAAACTAGGAGGCGTGTTCAGAGTACCGGACGAGCAAAAGCTGCTTTCGATGAGGACGGAAATATTTCTAATGTCAAAAGTGACACTGACAGAGATGCTGAAACGGTGACGCAAAATGAGGATGCTGATAGAAACAACAGTAGTAGAATTAACCTACAGGATATTTGCAAGGACTTGAACCAAGAAGGCGATGACCAGATGTTTGTTAGAAAAACATCAAGGGAAATTCAAGGACAAAATAGACGAAATCTTATTCACGAGGACATTGCTAAGGCAATAAAGCAACTTTGGAATAACGACAAAGGTATAAAGCAGTGTTTTGCACGTTCTAATGAGTTTCAATTGGAGGGCTCAGCTGCATACTACTTTGATAACATTGAGAAATTTGCTAGTCCGAATTATGTCTGTACGGATGAGGACATTTTGAAGGGCCGTATAAAGACTACAGGCATTACAGAAACCGAATTTAACATCGGCTCGTCCAAATTCAAGGTTCTCGACGCTGGTGGGCAGCGTTCTGAACGTAAGAAGTGGATTCATTGTTTCGAAGGAATTACAGCAGTTTTATTTGTTTTAGCAATGAGTGAATACGACCAGATGTTGTTTGAGGATGAAAGAGTGAACAGAATGCATGAATCAATAATGCTATTTGACACGTTATTGAACTCTAAGTGGTTCAAAGATACACCGTTTATTTTGTTTTTAAATAAAATTGATTTGTTCGAGGAAAAGGTAAAAAGCATGCCCATAAGAAAGTACTTTCCTGATTACCAGGGACGTGTCGGCGATGCAGAAGCGGGTCTAAAATATTTTGAGAAGATATTTTTGAGCTTGAATAAGACAAACAAACCAATCTACGTGAAACGAACCTGCGCTACCGATACCCAAACTATGAAGTTCGTATTGAGTGCAGTCACCGATCTAATCATCCAGCAAAACCTGAAA*GGTTGTGGCTTGTACTGA*gg**atcc**tgagacc | *GPA1-G(alpha)o* chimeric protein constructed by truncating the 5 C-terminal amino acids of *GPA1* (*S. cerevisiae)* and replacing these with those of human *G(alpha)o* (Brown *et al.* 2003). *GPA1* was amplified from genome of strain Design 4 and cloned into pYTK001 by BsaI digestion together with annealed oligos containing BsaI compatible overhangs. The resulting plasmid contains the gene with part 3 adaptors the MoClo cloning toolkit (Lee *et al.* 2015). |
| GPA1(11) | ggtctca**TATG**GGGTGTACAGTGAGTACGCAAACAATAGGTGACGAAAGTGATCCTTTTCTACAGAACAAAAGAGCCAATGATGTCATCGAGCAATCGTTGCAGCTGGAGAAACAACGTGACAAGAATGAAATAAAACTGTTACTATTAGGTGCCGGTGAGTCAGGTAAATCAACGGTTTTAAAACAATTAAAATTATTACATCAAGGCGGTTTCTCCCATCAAGAAAGGTTACAGTATGCTCAAGTGATATGGGCAGATGCCATACAATCAATGAAAATTTTGATTATTCAGGCCAGAAAACTAGGTATTCAACTTGACTGTGATGATCCGATCAACAATAAAGATTTGTTTGCATGCAAGAGAATACTGCTAAAGGCTAAAGCTTTAGATTATATCAACGCCAGTGTTGCCGGTGGTTCTGATTTTCTAAATGATTATGTACTGAAGTACTCAGAAAGGTATGAAACTAGGAGGCGTGTTCAGAGTACCGGACGAGCAAAAGCTGCTTTCGATGAGGACGGAAATATTTCTAATGTCAAAAGTGACACTGACAGAGATGCTGAAACGGTGACGCAAAATGAGGATGCTGATAGAAACAACAGTAGTAGAATTAACCTACAGGATATTTGCAAGGACTTGAACCAAGAAGGCGATGACCAGATGTTTGTTAGAAAAACATCAAGGGAAATTCAAGGACAAAATAGACGAAATCTTATTCACGAGGACATTGCTAAGGCAATAAAGCAACTTTGGAATAACGACAAAGGTATAAAGCAGTGTTTTGCACGTTCTAATGAGTTTCAATTGGAGGGCTCAGCTGCATACTACTTTGATAACATTGAGAAATTTGCTAGTCCGAATTATGTCTGTACGGATGAGGACATTTTGAAGGGCCGTATAAAGACTACAGGCATTACAGAAACCGAATTTAACATCGGCTCGTCCAAATTCAAGGTTCTCGACGCTGGTGGGCAGCGTTCTGAACGTAAGAAGTGGATTCATTGTTTCGAAGGAATTACAGCAGTTTTATTTGTTTTAGCAATGAGTGAATACGACCAGATGTTGTTTGAGGATGAAAGAGTGAACAGAATGCATGAATCAATAATGCTATTTGACACGTTATTGAACTCTAAGTGGTTCAAAGATACACCGTTTATTTTGTTTTTAAATAAAATTGATTTGTTCGAGGAAAAGGTAAAAAGCATGCCCATAAGAAAGTACTTTCCTGATTACCAGGGACGTGTCGGCGATGCAGAAGCGGGTCTAAAATATTTTGAGAAGATATTTTTGAGCTTGAATAAGACAAACAAACCAATCTACGTGAAACGAACCTGCGCTACCGATACCCAAACTATGAAGTTCGTATTGAGTGCAGTCACCGATCTAATCATCCAGCAAAACCTGAAA*GAATACAACCTATATTGA*gg**atcc**tgagacc | *GPA1-G(alpha)11* chimeric protein constructed by truncating the 5 C-terminal amino acids of *GPA1* (*S. cerevisiae)* and replacing these with those of human *G(alpha)11* (Brown *et al.* 2003). *GPA1* was amplified from genome of strain Design 4 and cloned into pYTK001 by BsaI digestion together with annealed oligos containing BsaI compatible overhangs. The resulting plasmid contains the gene with part 3 adaptors the MoClo cloning toolkit (Lee *et al.* 2015). |
| GPA1(q) | ggtctca**TATG**GGGTGTACAGTGAGTACGCAAACAATAGGTGACGAAAGTGATCCTTTTCTACAGAACAAAAGAGCCAATGATGTCATCGAGCAATCGTTGCAGCTGGAGAAACAACGTGACAAGAATGAAATAAAACTGTTACTATTAGGTGCCGGTGAGTCAGGTAAATCAACGGTTTTAAAACAATTAAAATTATTACATCAAGGCGGTTTCTCCCATCAAGAAAGGTTACAGTATGCTCAAGTGATATGGGCAGATGCCATACAATCAATGAAAATTTTGATTATTCAGGCCAGAAAACTAGGTATTCAACTTGACTGTGATGATCCGATCAACAATAAAGATTTGTTTGCATGCAAGAGAATACTGCTAAAGGCTAAAGCTTTAGATTATATCAACGCCAGTGTTGCCGGTGGTTCTGATTTTCTAAATGATTATGTACTGAAGTACTCAGAAAGGTATGAAACTAGGAGGCGTGTTCAGAGTACCGGACGAGCAAAAGCTGCTTTCGATGAGGACGGAAATATTTCTAATGTCAAAAGTGACACTGACAGAGATGCTGAAACGGTGACGCAAAATGAGGATGCTGATAGAAACAACAGTAGTAGAATTAACCTACAGGATATTTGCAAGGACTTGAACCAAGAAGGCGATGACCAGATGTTTGTTAGAAAAACATCAAGGGAAATTCAAGGACAAAATAGACGAAATCTTATTCACGAGGACATTGCTAAGGCAATAAAGCAACTTTGGAATAACGACAAAGGTATAAAGCAGTGTTTTGCACGTTCTAATGAGTTTCAATTGGAGGGCTCAGCTGCATACTACTTTGATAACATTGAGAAATTTGCTAGTCCGAATTATGTCTGTACGGATGAGGACATTTTGAAGGGCCGTATAAAGACTACAGGCATTACAGAAACCGAATTTAACATCGGCTCGTCCAAATTCAAGGTTCTCGACGCTGGTGGGCAGCGTTCTGAACGTAAGAAGTGGATTCATTGTTTCGAAGGAATTACAGCAGTTTTATTTGTTTTAGCAATGAGTGAATACGACCAGATGTTGTTTGAGGATGAAAGAGTGAACAGAATGCATGAATCAATAATGCTATTTGACACGTTATTGAACTCTAAGTGGTTCAAAGATACACCGTTTATTTTGTTTTTAAATAAAATTGATTTGTTCGAGGAAAAGGTAAAAAGCATGCCCATAAGAAAGTACTTTCCTGATTACCAGGGACGTGTCGGCGATGCAGAAGCGGGTCTAAAATATTTTGAGAAGATATTTTTGAGCTTGAATAAGACAAACAAACCAATCTACGTGAAACGAACCTGCGCTACCGATACCCAAACTATGAAGTTCGTATTGAGTGCAGTCACCGATCTAATCATCCAGCAAAACCTGAAA*GAATACAACCTAGTTTGA*gg**atcc**tgagacc | *GPA1-G(alpha)q* chimeric protein constructed by truncating the 5 C-terminal amino acids of *GPA1* (*S. cerevisiae)* and replacing these with those of human *G(alpha)q* (Brown *et al.* 2003). *GPA1* was amplified from genome of strain Design 4 and cloned into pYTK001 by BsaI digestion together with annealed oligos containing BsaI compatible overhangs. The resulting plasmid contains the gene with part 3 adaptors the MoClo cloning toolkit (Lee *et al.* 2015). |

**Table S4.** Strains used in this study.

| **Strain** | **Parental strain** | **Specifications** | **Origin** |
| --- | --- | --- | --- |
| yWS677 | BY4741 | *sst2Δ0 far1Δ0 bar1Δ0 ste2Δ0 ste12Δ0 gpa1Δ0 ste3Δ0 mf(alpha)1Δ0 mf(alpha)2Δ0 mfa1Δ0 mfa2Δ0 gpr1Δ0 gpa2Δ0* | (Shaw *et al.* 2019) |
| Design 4 | yWS677 | *ura3Δ0:pCCW12-STE2-tSSA1-pPGK1-GPA1-tENO2-pRAD27-LexA-PRD-tENO1-URA3; leu2Δ0:LexO(6x)-pLEU2m-sfGFP-tTDH1-LEU2* | (Shaw *et al.* 2019) |
| APS2 | Design 4 | *ura3Δ0 ste2Δ0* | This study |
| ASC2 | APS2 | *XI-2:LexO6xLEU2p-HIS3-tENO1-TEF2P-miRFP670-tADH1; XII-1:TEF1p-miRFP670-tCYC1* | This study |
| ASC4 | ASC2 | *gpa1Δ0* | This study |
| ASC4G0 | ASC4 | *XI-5:pPGK1-GPA1-tENO2* | This study |
| ASC4G0-1 | ASC4G0 | *XI-5:pPGK1-GPA1-tENO2; XII-2:pCCW12-ScSTE2-tSSA1* | This study |
| ASC4G1 | ASC4 | *XI-5:pPGK1-GPA1(i1)-tENO2* | This study |
| ASC4G2 | ASC4 | *XI-5:pPGK1-GPA1(i3)-tENO2* | This study |
| ASC4G3 | ASC4 | *XI-5:pPGK1-GPA1(o)-tENO2* | This study |
| ASC4G1h1 | ASC4 | *XI-5:pRPL18B-GPA1(i1)-tENO2* | This study |
| ASC4G1h2 | ASC4 | *XI-5:pTEF2-GPA1(i1)-tENO2* | This study |
| ASC4G1h3 | ASC4 | *XI-5:pTDH3-GPA1(i1)-tENO2* | This study |
| ASC4G9 | ASC4 | *XI-5:pPGK1-GPA1(q)-tENO2* | This study |
| ASC4G10 | ASC4 | *XI-5:pPGK1-GPA1(11)-tENO2* | This study |
| ASC4G1-3 | ASC4G1 | *XII-2:pCCW12-FFA2R-tSSA1* | This study |
| ASC4G2-3 | ASC4G2 | *XII-2:pCCW12-FFA2R-tSSA2* | This study |
| ASC4G3-3 | ASC4G3 | *XII-2:pCCW12-FFA2R-tSSA3* | This study |
| ASC4G1h1-3 | ASC4G1h1 | *XI-5:pRPL18B-GPA1(i1)-tENO2; XII-2:pCCW12-FFAR2-tSSA1* | This study |
| ASC4G1h2-3 | ASC4G1h2 | *XI-5:pTEF2-GPA1(i1)-tENO2; XII-2:pCCW12-FFAR2-tSSA1* | This study |
| ASC4G1h3-3 | ASC4G1h3 | *XI-5:pTDH3-GPA1(i1)-tENO2; XII-2:pCCW12-FFAR2-tSSA1* | This study |
| ASC4G9-3 | ASC4G9 | *XI-5:pPGK1-GPA1(q)-tENO2; XII-2:pCCW12-FFAR2-tSSA1* | This study |
| ASC4G10-3 | ASC4G10 | *XI-5:pPGK1-GPA1(11)-tENO2; XII-2:pCCW12-FFAR2-tSSA1* | This study |

**References**

Brown AJ, Goldsworthy SM, Barnes AA *et al.* The Orphan G Protein-coupled Receptors GPR41 and GPR43 Are Activated by Propionate and Other Short Chain Carboxylic Acids*. *Journal of Biological Chemistry* 2003;**278**:11312–9.

Hedin KA, Kruse V, Vazquez-Uribe R *et al.* Biocontainment strategies for in vivo applications of Saccharomyces boulardii. *Front Bioeng Biotechnol* 2023;**11**, DOI: 10.3389/fbioe.2023.1136095.

Lee ME, DeLoache WC, Cervantes B *et al.* A highly characterized yeast toolkit for modular, multipart assembly. *ACS Synth Biol* 2015;**4**:975–86.

Scott LH, Wigglesworth MJ, Siewers V *et al.* Genetically Encoded Whole Cell Biosensor for Drug Discovery of HIF-1 Interaction Inhibitors. *ACS Synth Biol* 2022;**11**:3182–9.

Shaw WM, Yamauchi H, Mead J *et al.* Engineering a model cell for rational tuning of GPCR signaling. *Cell* 2019;**177**:782-796.e27.
